# Primary orthologs from local sequence context

**DOI:** 10.1186/s12859-020-3384-2

**Published:** 2020-02-06

**Authors:** Kun Gao, Jonathan Miller

**Affiliations:** 1 School of Science, Southwest University of Science and Technology, 59 Qinglong Road, Mianyang, Sichuan Province 621010 People’s Republic of China; 2 Physics and Biology Unit, Okinawa Institute of Science and Technology Graduate University, 1919-1 Tancha, Onna-son, Kunigami-gun, Okinawa, 904-0495 Japan

**Keywords:** Primary/positional orthology, Genomic context, K-mer, Reciprocal best hit, Whole-genome alignment

## Abstract

**Background:**

The evolutionary history of genes serves as a cornerstone of contemporary biology. Most conserved sequences in mammalian genomes don’t code for proteins, yielding a need to infer evolutionary history of sequences irrespective of what kind of functional element they may encode. Thus, sequence-, as opposed to gene-, centric modes of inferring paths of sequence evolution are increasingly relevant. Customarily, homologous sequences derived from the same direct ancestor, whose ancestral position in two genomes is usually conserved, are termed “primary” (or “positional”) orthologs. Methods based solely on similarity don’t reliably distinguish primary orthologs from other homologs; for this, genomic context is often essential. Context-dependent identification of orthologs traditionally relies on genomic context over length scales characteristic of conserved gene order or whole-genome sequence alignment, and can be computationally intensive.

**Results:**

We demonstrate that short-range sequence context—as short as a single “maximal” match— distinguishes primary orthologs from other homologs across whole genomes. On mammalian whole genomes not preprocessed by repeat-masker, potential orthologs are extracted by genome intersection as “non-nested maximal matches:” maximal matches that are not nested into other maximal matches. It emerges that on both nucleotide and gene scales, non-nested maximal matches recapitulate primary or positional orthologs with high precision and high recall, while the corresponding computation consumes less than one thirtieth of the computation time required by commonly applied whole-genome alignment methods. In regions of genomes that would be masked by repeat-masker, non-nested maximal matches recover orthologs that are inaccessible to Lastz net alignment, for which repeat-masking is a prerequisite. mmRBHs, reciprocal best hits of genes containing non-nested maximal matches, yield novel putative orthologs, e.g. around 1000 pairs of genes for human-chimpanzee.

**Conclusions:**

We describe an intersection-based method that requires neither repeat-masking nor alignment to infer evolutionary history of sequences based on short-range genomic sequence context. Ortholog identification based on non-nested maximal matches is parameter-free, and less computationally intensive than many alignment-based methods. It is especially suitable for genome-wide identification of orthologs, and may be applicable to unassembled genomes. We are agnostic as to the reasons for its effectiveness, which may reflect local variation of mean mutation rate.

## Background

### Orthologs and paralogs

Sequences appearing in different genomes or within a single genome at frequencies beyond those expected on neutral evolution are expected to share common ancestors. Shared ancestry is known as *homology* and the corresponding genetic elements as *homologs* [1]. Homologs can be further classified as *orthologs* if they diverged via evolutionary speciation or *paralogs* if they diverged via duplication [2, 3]. Orthologs obtain special importance in phylogeny [3–6]: it is generally believed that orthologs from genomes of different species often if not always [7] share similar functions, while paralogs are more likely to develop new functions. Therefore, identifying orthologs among genomes of different species is fundamental to the fields of comparative Omics and is of great importance to our understanding of genome evolution and functional sequence innovation [6].

**Fig. 1:**
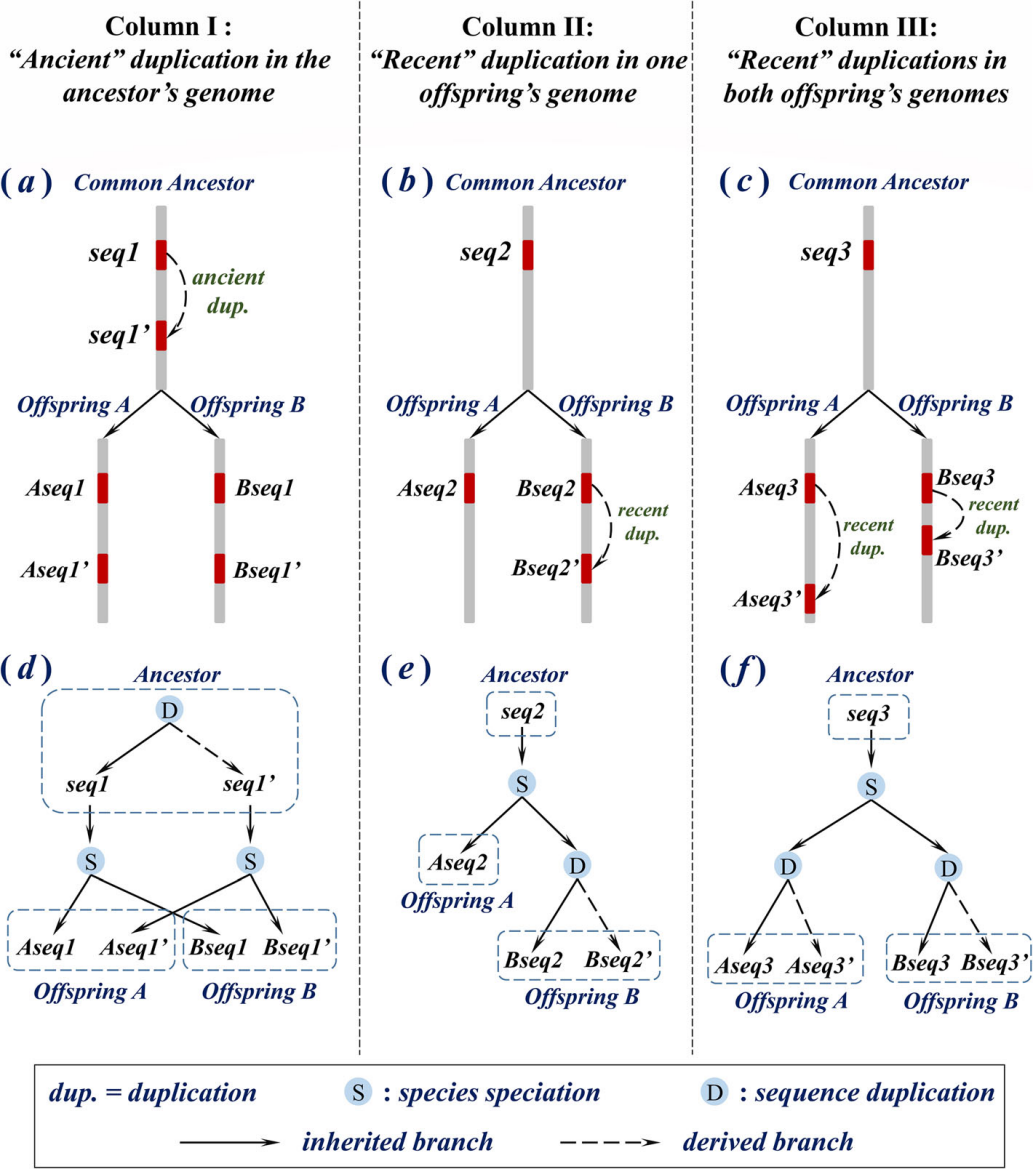
Emergence of homology classes distinguished by chronology of duplication and speciation events. **a**: An *ancient duplication* creates ***seq1’*** as a daughter copy of ***seq1*** in the ancestor’s genome. Then as the ancestor diverges into two offspring, ***A*** and ***B***, ***seq1*** and ***seq1’*** are inherited by both the offspring’s genomes. **b**: ***seq2*** of the ancestor’s genome is inherited by both the offspring’s genomes ***A*** and ***B***, then in the genome of ***B***, a *recent duplication* creates ***Bseq2’*** as a daughter copy of ***Bseq2***. **c**: similar to subfigure (**b**), only recent duplications in both genome ***A*** and genome ***B*** create ***Aseq3’*** and ***Bseq3’*** as daughter copies of ***Aseq3*** and ***Bseq3*** respectively. **d**-**f**: exhibit exactly the same evolutionary histories as subfigures (**a**)-(**c**) in gene trees. Dashed rectangles indicate genomes of different species. Solid edges (inherited branches) indicate evolutionary paths along which “inheritance between successive generations” applies. Dashed edges (derived branches) indicate creation of new duplicates. In this context, *the evolutionary path connecting a pair of primary orthologs must consist of inherited branches only*. Primary orthologs are conjectured to conserve their ancestral genomic positions, and to undergo constrained evolution. This figure can be compared with the figures in the Ensembl documentation page for “homology types” [8], and Fig. 1 in [9], whose “divergent edges” correspond to our “derived branches.”

In genome evolution, sequence duplication and other evolutionary events can complicate orthologous relationships. When *recent duplication* occurs in either genome subsequent to the branching of two genomes, new copies of a genomic sequence emerge as *in-paralogs* of the original. In terms of phylogeny, multiple in-paralogs in one genome should all correspond to the same ortholog in the other genome, so that orthology is not always one-to-one (see Fig. 1); such a relationship is called *co-orthology* [3, 6, 10]. In-paralogs do *not* necessarily all share the same function. The notion of co-orthology reflects an inconsistency in the customary application of the term “orthologs” as a synonym for “equivalent genes.”

### Concepts of primary ortholog

To overcome this inconsistency and further distinguish among co-orthologs those *isofunctional* [11] pairs of genes between the compared genomes—gene pairs more likely to play equivalent roles within both genomes than other homologs—different research groups have over the past decade applied a variety of methods to identify such orthologs. Terms to describe them include *true exemplar* [12], *main ortholog* [10, 13], *super ortholog* [14], *true ortholog* [15], *isoortholog* [16] and *positional ortholog* [17, 18]. The definitions of these terms are for the most part operational: they are implicitly defined by the computations and parameters used to determine them [19] and they are not fully consistent with one another. The dynamics of evolution of the corresponding orthologs are unfortunately not well reflected by these definitions; however, within recent literature the following three definitions stand out to us:

(i) The *primary ortholog* proposed by Han and Hahn [20] postulates that for a *recent* duplication, primary orthology only applies to the *original* (or *parent*) copy, but not to the *derived* (or *daughter*) copies. This definition has been accepted as a principle for identifying such orthologs, although the notions of “parent” and “daughter” copies of duplication remain without explicit definition, as their definitions are not fully articulated in [20].
(ii) The *positional ortholog* as redefined by Dewey [19] holds that such orthologs most faithfully reflect the *original positions* of their ancestral sequences within the common ancestor’s genome. Here the “original” and “derived” copies of duplication have been explicitly defined: in case of an *asymmetrical* duplication, removing the derived copies returns the genome to its previous state; however, in the event of *symmetrical* duplication positional orthologs are indistinguishable, and in the event of sequence transposition, positional orthology is disrupted.
(iii) The *primary ortholog* as redefined by Lafond et al. [9] denotes gene pairs that “have not been separated by an event of duplication followed by an increased rate of mutation.” This definition is based on an *asymmetrical evolution conjecture*: orthologs that conserved their ancestral genomic positions (e.g. parent copies of duplications) are “under greater evolutionary constraint than other homologs” [17, 19, 21, 22, 23, 24, 25, 26], whereas duplicates in non-ancestral positions (e.g. daughter copies of duplications) are “more likely to undergo positive selection” [27]. Lafond et al. also formalized a graphical description of primary orthologs: in a gene tree, paths between primary orthologs never contain a “divergent edge” (see [9] for more details).

### Classifying orthologs

Orthologs are customarily identified through two classes of methods: phylogeny-based and similarity-based. Phylogenetic analysis of gene lineage is thought in principle to enable the strongest discrimination between orthologs and paralogs [28], but fails to distinguish primary/positional orthologs from other orthologs. Similarity-based methods tend to neglect non-primary/non-positional orthologs as well as paralogs, whereas they can be expected to identify primary/positional orthologs. A standard similarity-based method assumes that orthologs are most likely to be those homologs that diverged least within a given genome pair [6, 9]; however, when asymmetrical evolution does *not* apply, this method may not work well for co-ortholog identification: for homogeneous variation rates, all those in-paralogs that branched simultaneously from their orthologous cognates in another genome are expected to exhibit comparable sequence divergence. Thus, sequence similarity *within homologous regions* is not always sufficient to distinguish different classes of co-orthologs from one another.

### Genomic context

A variety of methods have invoked *genomic context* to predict or refine orthologous relationships [19]. It has been observed that in-paralogs at different locations within a genome are likely to exhibit differences in their *neighborhoods*: parent and daughter copies of a duplication, while similar to one another in sequence content, are often embedded into different genomic contexts that did not themselves *also* undergo duplication at the same time. Here the general notion of “genomic context” provides a tool to differentiate primary/positional orthologs from all other homolog pairs. In previous literature, *synteny information*—in the sense of “conserved gene order”—is often taken as an indicator of genomic context that is incorporated into methods based primarily on sequence similarity or on gene evolution models [13, 19].

### Inherited and derived branches

In this paper, we further develop genomic context as a tool to distinguish between parent and daughter copies of duplication. We propose and validate a new method of inferring “primary orthologs” from whole genome nucleotide sequences. Our notion of sequence homology extends naturally from protein-coding *genes* addressed in most previous literature, to genomic sequences *in general*. As in [9], we reformulate the definition of “primary orthologs” from the perspective of a phylogenetic tree. The following discussion is illuminated by Fig. 1, which exhibits the emergence of distinct classes of homology subject to different evolutionary histories of sequence duplication and species speciation; the corresponding classification of homologs is shown in Table 1. A pair of genomic sequences is designated a pair of primary orthologs if both of them are connected to a same most recent common ancestral sequence in a phylogenetic tree solely through *inherited branches*: branches along which inheritance from parent to offspring applies. Orthologs that are not primary are called “secondary orthologs” [20]. We postulate that “inheritance” occurs only between successive generations: sequence duplication is not considered “inheritance.” Therefore, within a phylogenetic tree, all branches subsequent to a speciation node are taken as inherited branches. However, for each node representing a duplication event, only its parent branch, which is unique for each duplication node, is an inherited branch. All daughter branches are called *derived branches* (essentially equivalent to the “divergent edges” of [9]). As in [9], primary orthology is a transitive relation, and the evolutionary path that connects a pair of primary orthologs in a phylogenetic tree must consist of inherited branches only.

**Table 1:** Relationship of different types of homologs in Fig. 1

Homologs	Column I	Column II	Column III
*Primary orthologs*	(*Aseq1*, *Bseq1*) (*Aseq1’*, *Bseq1’*)	(*Aseq2*, *Bseq2*)	(*Aseq3*, *Bseq3*)
*Secondary orthologs*		(*Aseq2*, *Bseq2’*)	(*Aseq3*, *Bseq3’*) (*Aseq3’*, *Bseq3*) (*Aseq3’*, *Bseq3’*)
*In-paralogs*	(*Aseq1*, *Aseq1’*) (*Bseq1*, *Bseq1’*)	(*Bseq2*, *Bseq2’*)	(*Aseq3*, *Aseq3’*) (*Bseq3*, *Bseq3’*)
*Out-paralogs*	(*Aseq1*, *Bseq1’*) (*Aseq1’*, *Bseq1*)		

### Primary orthologs

The above definition of primary ortholog is essentially a hybrid of “positional ortholog” as defined in [19] and “primary ortholog” as defined in [9]. We assume that parent copies of duplications maintain longer stretches of matches within their flanking regions, while their daughter copies solely exhibit shorter matches that contribute only to the duplicated segments (see Methods for details); this idea is similar to that in [20]. Since parent copies of duplication are conjectured either to conserve their ancestral genomic positions in [19] or to undergo a lower variation rate than their daughter copies in [9], our assumption remains tenable provided either or both of these conditions [9, 19] apply: *Orthologs that have either conserved their ancestral positions in the genomes or have undergone a lower rate of variation than other homologs are taken as primary orthologs*. The method we propose below identifies “primary orthologs” that can be either positional or non-positional. In practice, we infer primary orthologs and other homologs genome-wide by exploiting local contexts of matches at scales much shorter than a gene, whereas most previous methods, including those in [19, 20], rely on conserved synteny or large regions of colinearity over relatively long domains that often span a series of genes.

The paper is organized as follows: in Methods, we introduce a sequence-based property of *nesting* among maximal matches, demonstrate its relationship to primary/positional orthology, and propose a new context-based method to identify such orthologs for a pair of genomes. In Results, we quantify its effectiveness: firstly, we test it under a neutral model of genome evolution by numerical simulation, then, we compare primary orthologous genes in natural genomes inferred by our method with those inferred by BLAST RBHs, and with those annotated by Ensembl Compara.

## Methods

### Definition of nested and non-nested maximal matches

#### Nesting

In this paper, a genomic *sequence* refers to a constituent string of bases *and* its position within the genome. In contrast, the term *string* refers solely to the series of nucleotides irrespective of position in the genome. Two genomic sequences are to be thought of as “the same sequence” only if they occupy the same site in a genome: they share not only the same content of string but also the same coordinates in the genome. As defined in [29], a genomic sequence is thought of as *nested* in another genomic sequence of the same genome, only if the region of genome covered by the first sequence is a proper subinterval of the region covered by the second sequence.

#### Maximal matches

**Fig. 2:**
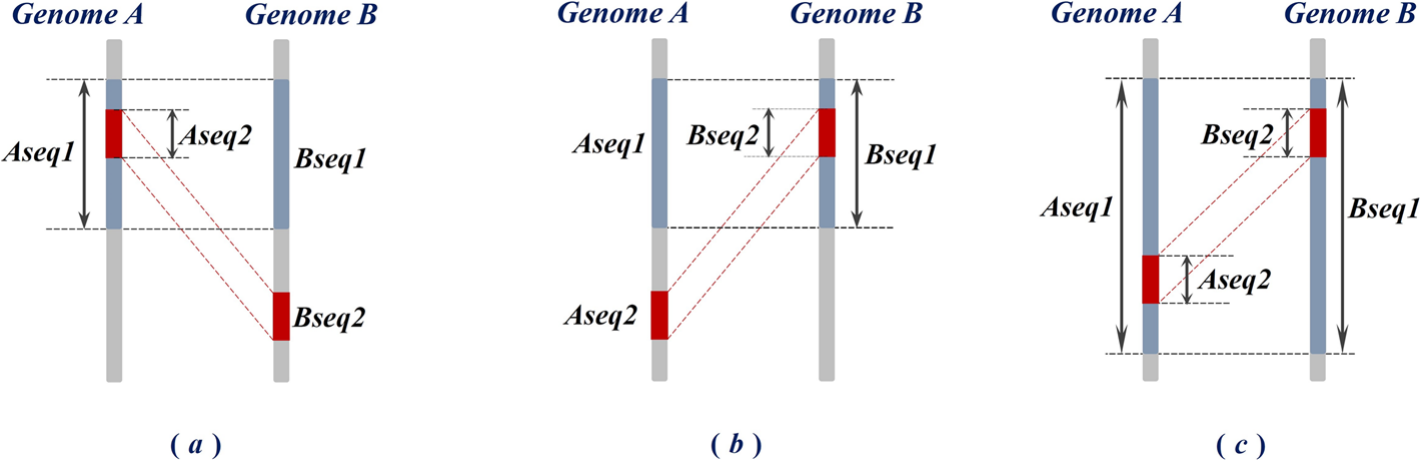
Relationship of *nesting* between maximal matches. In each subfigure, (***Aseq1***, ***Bseq1***) and (***Aseq2***, ***Bseq2***) are two pairs of maximal matches; (***Aseq2***, ***Bseq2***) is nested in (***Aseq1***, ***Bseq1***), since either—as in subfigure (**a**) and (**b**)—or both—as in subfigure (**c**)—sequences of (***Aseq2***, ***Bseq2***) are nested in the corresponding sequences of (***Aseq1***, ***Bseq1***)

We now generalize the concept of “nesting” from single sequences to sequence pairs. We define a ***maximal match*** between two genomes as a contiguous run of matching bases that is extendable at neither end. It is represented as a pair of highly similar (or identical) strings, one in each genome, and terminated by mismatches on both ends. A maximal match is said to be *nested* in another maximal match if *either* sequence constituting the first maximal match is nested in the corresponding sequence of the second maximal match; see Fig. 2. Based on the above definition, all maximal matches between two genomes can be classified into two mutually exclusive groups: if a maximal match is nested in another maximal match, it is classified as a ***nested maximal match***. For example, (***Aseq2***, ***Bseq2***) in Fig. 2 represent a nested maximal match. If a maximal match is not nested in *any other* maximal match, it is a ***non-nested maximal match***.

Note that in the above definition, strings of a maximal match are not required to be identical to each other; stringency of a “match” can be relaxed. We call maximal matches with *identical* strings maximal ***exact matches***; alternatively maximal matches can be defined with relaxed matching stringencies; see [30] for some examples. In this paper, our non-nested and nested maximal matches, unless indicated otherwise, are all maximal *exact* matches and we call them ***non-nested*** and ***nested exact matches***. However, in our numerical simulations in below, to account for the impact of point mutations we also consider non-nested and nested maximal matches yielded by *inexact* matches; see Methods for the definition of *non-nested and nested EDMs*.

### Identifying primary orthologs by non-nested maximal matches

**Fig. 3:**
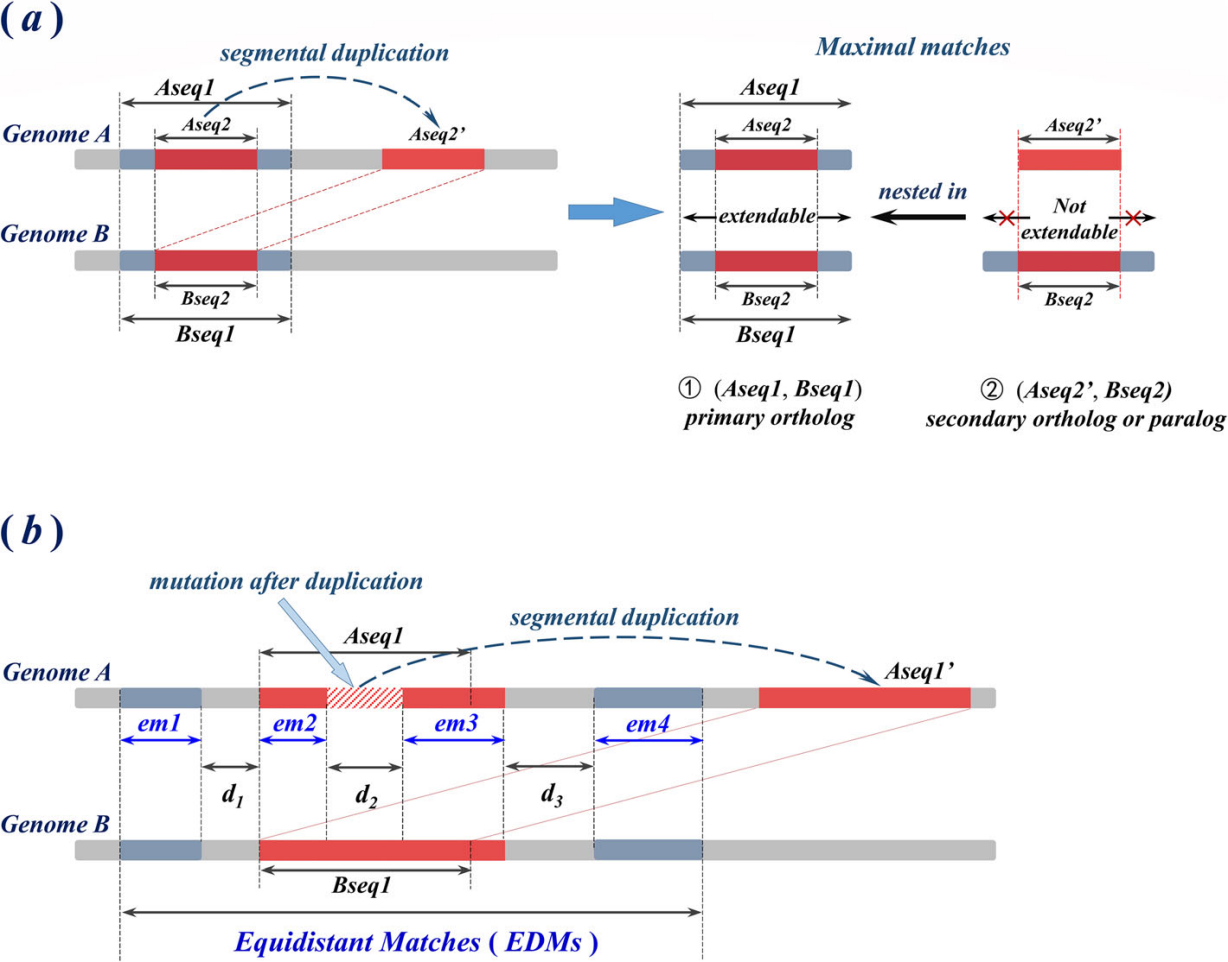
Secondary orthologous and paralogous regions tend to be nested in primary orthologous regions. Blue and red bars in each subfigure indicate regions of homology between genomes ***A*** and ***B***, and dashed arrows indicate the direction of segmental duplications, which could be *either recent or ancient*. **a** When the parent copy of duplication is well conserved, our proposal applies on ***exact matches***. Comparing with an outgroup sequence ***Bseq2*** in a different genome, the exact match between the parent copy ***Aseq2*** and ***Bseq2*** can usually be extended into the flanking region until the extended match (***Aseq1***, ***Bseq1***) is maximal. In contrast, the exact match between the daughter copy ***Aseq2’*** and ***Bseq2*** is already a maximal match, and is not extendable. As a result, the latter exact match (which is secondary orthologous or paralogous) is ***nested*** in the former (which is primary orthologous). **b** When the parent copy of duplication is degraded by mutations after duplication, our method based on exact matches may yield misclassifications between primary orthologs and other homologs. The shaded region in subfigure (**b**) represents a region of mutation with length ***d***_***2***_ that breaks down the primary orthologous region (***Aseq1***, ***Bseq1***) into two shorter exact matches, ***em2*** and ***em3***. To compensate for the impact of such mutations, we concatenate neighboring exact matches (***em1***~***em4*** in subfigure (**b**)) separated by regions of mismatches whose lengths in both genomes coincide, into a longer matched region, which we call an “equidistant match” (*EDM*). With *EDMs*, the secondary orthologous (or paralogous) region (***Aseq1’***, ***Bseq1***) is still nested in the region of *EDMs*, which is primary orthologous

We anticipate that primary orthologs can be distinguished from secondary orthologs and paralogs through their associated non-nested and nested maximal matches respectively. We observe that secondary orthologous and paralogous regions are often nested in primary orthologous regions (see Fig. 3), so that *nested maximal matches are associated chiefly with secondary orthologs and paralogs, whereas non-nested maximal matches are associated chiefly with primary orthologs.*

#### Non-nested exact matches (NNEM)

Figure 3 (a) illustrates the above proposal with a representative example. ***Aseq2***, which is a proper subsequence of ***Aseq1*** in ***genome A***, is duplicated into a daughter copy, ***Aseq2’***. ***Bseq1*** and ***Bseq2*** in ***genome B***, are respectively the primary orthologous counterparts of ***Aseq1*** and ***Aseq2***. With respect to the date of speciation of genomes ***A*** and ***B***, the *exact match* between ***Aeq2’*** and ***Bseq2*** constitutes a pair of secondary orthologs if the duplication is *recent*, or it constitutes a pair of out-paralogs if the duplication is *ancient*. Since primary orthologs are conjectured to both conserve their ancestral positions in the genome, ***Aseq2*** and ***Bseq2*** are expected to share not only the same string, but also the same genomic context, whereas ***Aseq2’*** is expected to exhibit a distinct context. As a result, the exact match between ***Aseq2*** and ***Bseq2*** is probably not maximal: it most likely extends into the flanking regions (blue regions in Fig. 3 (a)), until local sequence variations terminate the match on both ends. In contrast, distinct contexts prevent the match between ***Aseq2’*** and ***Bseq2*** from extending further; in most cases this maximal match is constituted solely by the duplicated region (red regions in Fig. 3 (a)), so that secondary orthologous or paralogous region (***Aseq2’***, ***Bseq2***) is nested in the primary orthologous region (***Aseq1***, ***Bseq1***). (***Aseq2’***, ***Bseq2***) constitute a pair of nested exact match, whereas (***Aseq1***, ***Bseq1***) constitute a pair of non-nested exact matches.

#### Non-nested equidistant matches (non-nested EDM)

Orthologous regions may undergo sequence variation subsequent to duplication that yields misclassification between primary orthologs and other homologs if the variation takes place within the parent copy of the duplication. Figure 3 (b) shows an example: after ***Aseq1*** has been duplicated into ***Aseq1’***, a subsequent variation of length ***d***_***2***_ (shaded region in Fig. 3 (b)) emerges within the region of ***Aseq1*** and breaks the exact match between ***Aseq1*** and ***Bseq1*** into two shorter exact matches, ***em2*** and ***em3***. Although these shorter exact matches are by definition primary orthologous, ***em2*** is nested in the secondary orthologous or paralogous region (***Aseq1’***, ***Bseq1***), whereas ***em3*** partly overlaps with the latter. As a result, (***Aseq1’***, ***Bseq1***) is identified as a pair of non-nested exact match; under the effect of variation after duplication, non-nested exact matches may not well distinguish primary orthologs from other homologs.

To better account for such variations we can incorporate *inexact matches*. If genomic sequences constituting neighboring exact matches are separated by regions of mismatch whose lengths in both genomes coincide, and provided that the length of the mismatched region does not exceed a given threshold, we concatenate these neighboring exact matches—including the mismatches between them—into an “equidistant match” (*EDM*). For example in Fig. 3 (b), two exact matches, ***em1*** and ***em2***, are separated by a region of mismatch with length ***d***_***1***_ in both ***Genome A*** and ***Genome B***, and so are ***em2*** and ***em3*** by a region of length ***d***_***2***_, and ***em3*** and ***em4*** by a region of length ***d***_***3***_. If all these lengths ***d***_***1***_, ***d***_***2***_ and ***d***_***3***_ do not exceed a predetermined threshold, we concatenate ***em1~em4*** into a single EDM. Non-nested and nested maximal matches are subsequently classified among all EDMs as in Fig. 2; we call such non-nested and nested maximal matches respectively *non-nested EDM* and *nested EDM*. EDMs overcome the impact of local mutations; with EDMs, secondary orthologous and paralogous regions are still nested in primary orthologous regions, even if the parent copies of duplications are not always well conserved; see Fig. 3 (b).

### Genome intersection

To obtain all non-nested and nested maximal matches between two genomes, we first identify all maximal matches between these genomes. This requires a genome comparison procedure; most generally, a *genome alignment*. Our non-nested and nested maximal matches are obtained without loss of generality from a special type of genome alignment, which we call *genome intersection*. We define an *intersection* between two genomes as *a complete set of maximal matches* that exhaustively recovers all sequences shared between these two genomes. Intuitively speaking, we view a genome as a “bag of sequences” labeled by their coordinates, and extract all sequences that occur in both genomes at least once, recording strings and positions. Non-nested and nested maximal matches are obtained from the intersection afterwards (see Methods and Additional file 1: supplementary material 1 for computational details).

In contrast to traditional alignment methods that are only *algorithmically* defined, genome intersection is *explicitly* defined, and nearly parameter-free: the only parameter involved in an intersection is the minimal length of maximal matches extracted. For mammalian genomes, this minimal length is chosen as 30~50 nucleotide bases; setting a greater value for this minimal length may enhance the plausibility of homology predicted by the intersection (i.e., the *precision* of the method), but at the same time diminish the coverage of the intersection over all homologs (i.e., the *recall rate* of the method).

Exhaustive whole genome intersection was at one time computationally challenging, but with existing hardware technology and computing algorithms, whole-genome intersection can be readily performed with a variety of software, many of which are based on a *suffix tree* or *suffix array*, for example, *SEQANALYSIS* [29, 31] and *MUMmer* [32, 33, 34, 35]. Such data structures can be built and searched in linear time and linear space. Without loss of generality we obtain our intersections with *SEQANALYSIS*; nevertheless, our method can also be implemented with a traditional alignment. For example, non-nested and nested maximal matches identified in a Lastz raw alignment are referred to as “*non-nested and nested CMRs*,” and their utility has been demonstrated in [36].

### Nested and non-nested maximal matches in whole-genome intersection

*SEQANALYSIS* organizes its output in a compact way by collating each sequence shared between the compared genomes into a *maxmer* indexed by its string. A maxmer is defined *not* merely as a string, but rather as a string *together with* a list of positions/coordinates indicating *where* that string is found within the compared genomes. Each position/coordinate denotes an *occurrence* of that string. Different maxmers correspond to different strings. Each occurrence of a maxmer string in one genome constitutes a match, but not necessarily a *maximal* match, to each occurrence of that string in the other genome. For each maxmer, there is at least one pair of occurrences—one occurrence from each genome—that together constitute a maximal match (see details in [29]).

*SEQANALYSIS* provides an expedient way to quickly identify non-nested and nested maximal matches, classifying each maxmer as *super* or *local*. A maxmer is a *super maxmer* if its string is *not* a proper substring of the string of any other maxmer; otherwise it is a *local maxmer*. We call a super maxmer *unique* if it contains exactly one occurrence in each of the compared genomes. On the other hand, occurrences of *local* maxmers can be subclassified as *nested* or *non-nested* according to the genomic regions they cover: a *non-nested occurrence* covers a region of genome that is *not* a proper subinterval of any region covered by an occurrence of any other maxmer; otherwise the occurrence is *nested*; for details see [29, 37]. By definition, our non-nested maximal matches consist of super maxmers or non-nested occurrences of local maxmers; conversely, a maximal match is nested only if at least one of the two sequences that constitute it is a nested occurrence of a local maxmer; see Table 2 for the correspondence. This classification allows us to identify non-nested and nested maximal matches independently (see Additional file 1: supplementary material 1 for details) and greatly reduces the computational burden.

**Table 2:** Correspondence among combinations of maxmer occurrences and non-nested/nested maximal matches. “Non-nested and nested occurrences” of local maxmers in [29, 37] differ from “non-nested and nested maximal matches” as we define in this table: an occurrence is a single sequence, whereas a maximal match consists of a pair of sequences. Maxmers are classified as “super” or “local.” Occurrences of super maxmers are subclassified as *unique super*, if they have exactly one occurrence in each of the corresponding genomes; or *non-unique super*, if occurrences appear multiply in at least one of those genomes. Occurrences of local maxmers are subclassified as *non-nested* or *nested* as in [29]. The designations *super* and *local* are exclusive of each other, therefore these four types of maxmer occurrences yield six different combinations, of which four are non-nested maximal matches and two are nested maximal matches

*Occurrences* of maxmers	super maxmer	(a) *unique super* or (b) *non-unique super*
	local maxmer	(c) *non-nested local* or (d) *nested local*
Non-nested maximal matches	*MUMs*	(1) two *unique supers*
	*Non-nested maximal matches* but not *MUMs*	(2) a *unique super* + a *non-unique super* (3) two *non-unique supers* (4) two *non-nested locals*
Nested maximal matches		(5) a *non-nested local* + a *nested local* (6) two *nested locals*, if together they constitute a maximal match

For this paper, we retrieve with *SEQANALYSIS* all occurrences of “4-base maxmers” from the compared genomes; the latter were *not* preprocessed to censor or redact repeats [38]. Non-nested and nested *exact matches* are obtained from different combinations of the occurrences of these maxmers according to Table 2; see Additional file 1: supplementary material 1 for computational details. *SEQANALYSIS* also provides an option –*sp* to output all EDMs with a given maximal length of mismatches, and we wrote a simple perl script to obtain non-nested EDMs for our numerical simulation. Alternatively, one can retrieve another set of maximal matches, maximal unique matches (“*MUMs*”, see (1) in Table 2), which requires uniqueness of each sequence: exactly one occurrence in each of the compared genomes. *MUMs* can either be identified by *SEQANALYSIS* according to Table 2, or directly output by *MUMmer* with switch *–mum*. *MUMs* form a *proper subset* of all non-nested maximal matches defined in this paper (see Table 2); thus they can also be taken as candidates for primary orthologs, and all calculations in this paper can be carried out on *MUMs*. Compared to non-nested maximal matches, *MUMs* tend to exhibit higher precision, but lower recall rate for primary ortholog identification. For closely related genomes our experience suggests non-nested maximal matches as candidates for primary orthologs; however, for more distantly related genomes *MUMs* may yield better performance; see Additional file 1: Figure S7 for their applications.

In principle different pairs of primary orthologs should not overlap, but because of sequence variation non-nested maximal matches that are misclassified as in Fig. 3 (b) may overlap with one another. Overlaps among non-nested maximal matches can be used as an indicator of the errors in our identification; they can be removed to improve the identification precision. For our calculations we have removed all the overlaps among non-nested maximal matches.

### Reciprocal best hits (RBHs) of genes determined by a given group of maximal matches

**Fig. 4:**
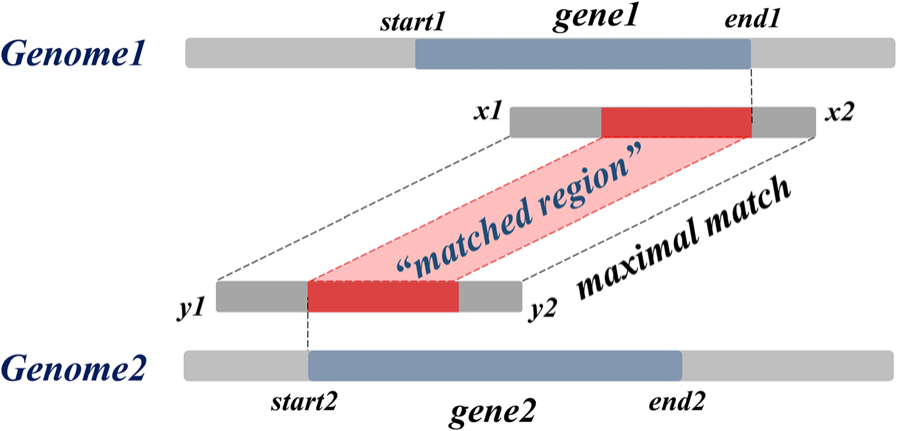
Matched region between a pair of genes associated to a given maximal match

Given the coordinates of genes within the genome, it is straightforward to measure the overlap between a maximal match and a pair of homologous genes: as in Fig. 4, ***gene1*** occupies a region [*start1*, *end1*] in ***Genome1***, and ***gene2*** occupies a region [*start2*, *end2*] in ***Genome2***. A maximal match consisting of region [*x1*, *x2*] of ***Genome1*** and [*y1*, *y2*] of ***Genome2***, overlaps with these two genes. We define a pair of subsequences with equal lengths, [*max*{*x1*, *start1*, *start2*+*x1*-*y1*}, *min*{*x2*, *end1*, *end2*+*x2*-*y2*}] in ***Genome1*** and [*max*{*y1*, *start2*, *start1*+*y1*-*x1*}, *min*{*y2*, *end2*, *end1*+*y2*-*x2*}] in ***Genome2*** as the *matched region* between ***gene1*** and ***gene2*** *associated to this single maximal match* (red bars in Fig. 4).

For a given pair of genes, we define a “hit score” by the sum of lengths of all matched regions between this pair of genes, associated to a selected group of maximal matches. For each gene in one genome, the gene in the other genome that shares the greatest hit score with it is defined as its (single-directional) best hit. If two genes are mutual best hits, then this pair of genes is defined as a *reciprocal* (or *bidirectional*) *best hit* of genes, *determined by the given group of maximal matches*. To distinguish our definition of reciprocal best hit from the conventional use of the acronym “RBH” that refers to BLAST RBH [39, 40, 41, 42, 43, 44], we call the RBHs defined in this paper ***maximal-match RBHs*** (or *mmRBHs* for short). mmRBHs depend on the specific group of maximal matches that determine them. In this paper we take the mmRBHs determined by *non-nested exact matches*, which we call ***non-nested RBHs***, as candidates for primary orthologous genes between the compared genomes, using as a reference those determined by *all* maximal matches between the same pair of genomes.

For comparison, we also retrieve the widely-used BLAST RBHs: we retrieve in the following sections the BLAST RBHs for the nucleotide sequences in our simulation, and the BLAST RBHs of genes between compared genomes. We observe that more than half of the genes in our calculation are not protein-coding; to cover all sequences and genes we implement BLAST search on nucleotide sequences by Blastn, and retrieve the reciprocal best hits by the alignment score.

## Results

### Numerical simulations: performance of our method on the nucleotide level

To evaluate the method proposed above, we implement a series of numerical simulations. We simulate evolution of genomes with two basic processes, segmental duplication and point mutation. In this paper, by “point mutation” we refer to single-base substitution. With a neutral evolution model [45] studied by Massip and Arndt [46, 47], we create a synthetic sequence of length *L* and take it as a common ancestral genome. This genome, as demonstrated by Massip and Arndt, contains repetitive sequences whose length distribution resembles those observed in natural genomes [30, 46, 47]; in our subsequent simulations, these repetitive sequences play the role of ancient duplications.

We follow the same dynamics once the ancestral genome diverges into two independent lineages. For each lineage, we simulate its subsequent evolution by implementing the same model continuously on its genome, and duplications introduced to the genomes therein simulate recent duplications. Following [46, 47], at each time step we choose at a random position in the genome a subsequence of length *K*. We duplicate the string of this subsequence and let it substitute for another subsequence of the same length at another random position in the same genome to maintain constant genome size (as in [46, 47]). Simultaneously, we randomly mutate a series of nucleotide bases at randomly chosen sites; the numbers of genome bases varied by point mutation and by segmental duplication constitute a fixed ratio *μ*/*ν*, where *μ* and *ν* represent mutation rate and duplication rate respectively, both weighted by length of bases. After a stationary state is obtained, we run the model independently and with the same parameters on each of the diverged lineages. During the above process, we record the ancestral positions of all sequences, so that parent and daughter copies of all duplications can be differentiated, and so that primary orthologs between the diverged lineages are readily distinguished. Over the course of the simulation, we compare the genomes of the diverged lineages at different evolutionary distances by intersection; identify all non-nested maximal matches according to Table 2; and evaluate the outcome by calculating *precision* and *recall*:

$$ Precision=\frac{\#\left(\ \mathrm{non}-\mathrm{nested}\ \mathrm{maximal}\ \mathrm{matches}\ \mathrm{that}\ \mathrm{are}\ \mathrm{primary}\ \mathrm{orthologs}\right)}{\#\left(\mathrm{all}\ \mathrm{non}-\mathrm{nested}\ \mathrm{maximal}\ \mathrm{matches}\right)}, $$

$$ Recall=\frac{\#\left(\ \mathrm{primary}\ \mathrm{orthologs}\ \mathrm{that}\ \mathrm{are}\ \mathrm{non}-\mathrm{nested}\ \mathrm{maximal}\ \mathrm{matches}\ \right)}{\#\left(\mathrm{all}\ \mathrm{primary}\ \mathrm{orthologs}\right)}, $$

where #() represents the *total number of bases* contained in the indicated set of maximal matches. Computing precision and recall instead by the *number of maximal matches* yields only minor differences.

### Parameters of the simulation

**Fig. 5:**
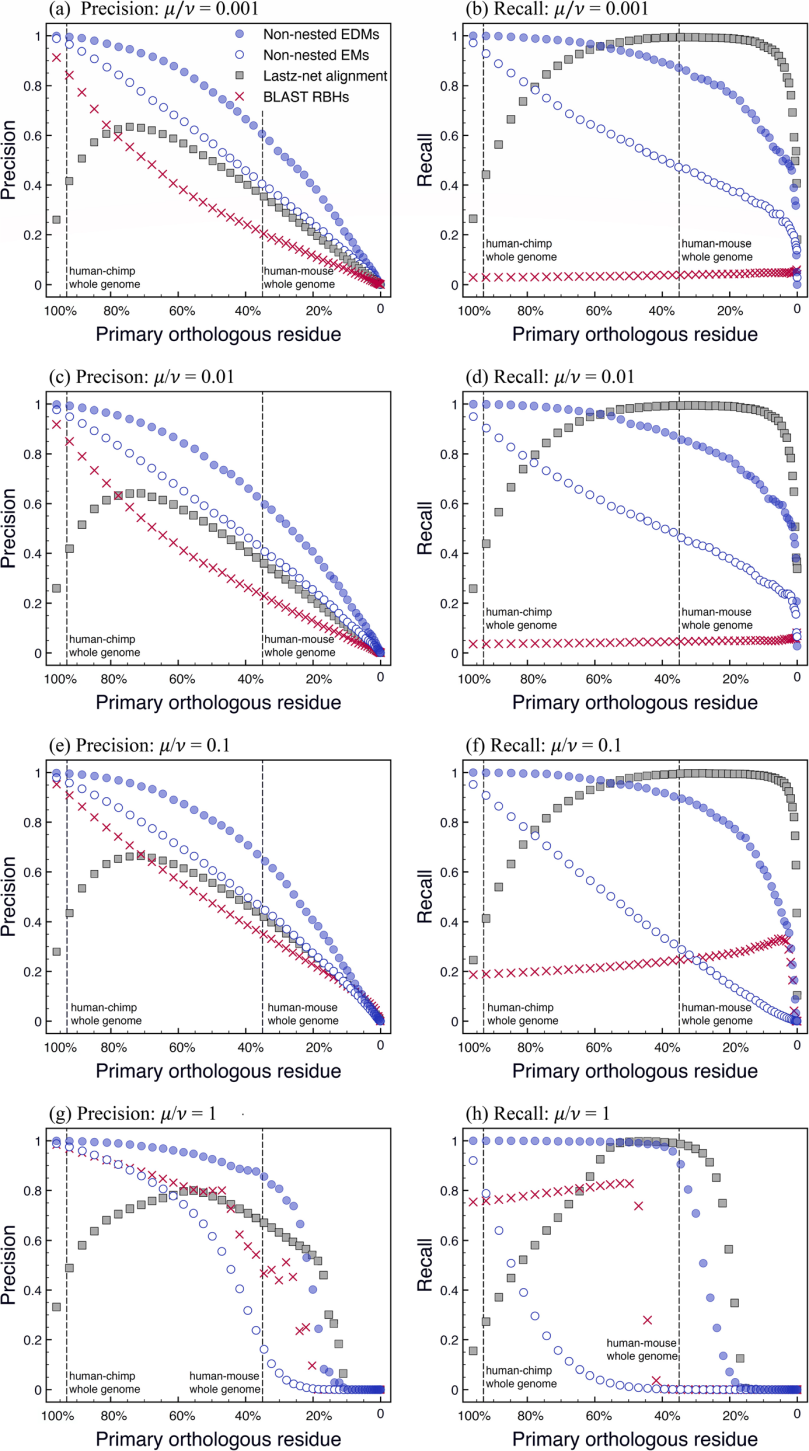
Precision and recall of our method derived from numerical simulations. The x-axis shows the proportion of genomes covered by *primary orthologous residue*: genome bases that are occupied by conserved primary orthologs, indicating a measure of the evolutionary distance between the diverged lineages. Vertical dashed lines indicate the evolutionary distances estimated for the human-chimpanzee (~ 93%) and human-mouse (~ 35%) genomes. Different subfigures show the simulations under different mutation-duplication ratio *μ*/*ν*: (**a**)-(**b**) *μ*/*ν* = 0.001; (**c**)-(**d**) *μ*/*ν* = 0.01; (**e**)-(**f**) *μ*/*ν* = 0.1; and (g)-(h) *μ*/*ν* = 1. In different subfigures, we use the same symbols to represent the same datasets: solid squares for Lastz net alignment, crosses for BLAST RBHs, hollow circles for non-nested exact matches (“non-nested EMs”), and solid circles for “non-nested EDMs”. For the latter, the minimal length of a single exact match within each EDM is set to 20 bases for all subfigures, and the maximum length of a single run of contiguous mismatches is set to *d* nucleotide bases: *d* = 2 for subfigures (**a**)-(**b**), *d* = 5 for (**c**)-(**f**), and *d* = 15 for (**g**)-(**h**). Curves in the figure are obtained by averaging over 10 realizations; the same curves with standard deviations indicated by error bars are shown in Additional file 1: Figures S3, S4 and S5

The above simulation depends on three parameters: the genome size *L*, the duplication length *K*, and the ratio of mutation rate *μ* over duplication rate *ν*, both weighted by length of bases. As demonstrated by [46] the − 3 power-law tail of the length distribution of repetitive sequences relies weakly on the parameters; different parameter values alter the amplitude, but not the form of the length distribution; however, the performance of our ortholog identification method may depend critically on these parameters. Therefore, in the simulation shown in Fig. 5 we tried to set parameter values comparable to those of natural genomes, if known. We take *L* = 10^8^ nucleotide bases, which is to the same length as a single chromosome of a mammalian genome, and *K* = 5☓10^4^ nucleotide bases, which is the longest duplication length in a real genome [30]. Following [46, 47] we carry out our simulations with four different *μ*/*ν* ratios: subfigure (a)-(b) for *μ*/*ν* = 0.001, (c)-(d) for *μ*/*ν* = 0.01, (e)-(f) for *μ*/*ν* = 0.1, and (g)-(h) for *μ*/*ν* = 1. Note that our duplication rate *ν* is weighted by length of bases, and the *μ*/*ν* ratios selected in our simulation are consistent with those applied in [46, 47].

To validate our choice of parameters, we estimate the *μ*/*ν* ratio of real genomes by fitting the match length distributions (MLD as in [46]) of our synthetic sequences to the MLD of real genome sequences, see Additional file 1: Figure S1 and S2. For each real genome sequence, we create synthetic sequences of the same length with different *μ*/*ν* ratios; the duplication length *K* for these synthetic sequences is taken as the longest match length within the given real genome sequence. Then we estimate the *μ*/*ν* ratio for the real genome sequence by comparing its MLD to the MLD of the synthetic sequences with different *μ*/*ν* ratios. For example, in Additional file 1: Figure S1 (a), the MLD of the human protein-coding sequences overlaps well with the MLD of synthetic sequence with *μ*/*ν* = 1, suggesting that the *μ*/*ν* ratio within the protein-coding regions of the human genome is close to 1. Additional file 1: Figure S1 (b) suggests that the *μ*/*ν* ratio within protein-coding regions of the mouse genome is close to 0.1. These estimations are comparable to those from previous literature. In [48] Lynch and Conery estimated that gene duplication arises at an approximate rate of 1 per gene per 100 million years in eukaryotes such as human and mouse. On the other hand, the estimated mutation rate depends on the accuracy and reliability of molecular clock, and on the genes and species that are analyzed [49, 50, 51]. In previous literature, mutation rate estimated for vertebrates genes varies from 10^− 9^ per site per year to 10^− 8^ per site per year [50, 51, 52, 53]. Thus the *μ*/*ν* ratio for protein-coding regions of vertebrates genomes should be between 0.1 and 1, which is of the same order of magnitude as our estimates in Additional file 1: Figure S1 (a) and (b).

Similarly, we can estimate the *μ*/*ν* ratios for whole-genome sequences, both protein-coding and non-protein-coding. In this paper, we study all sequences of genomes rather than protein-coding genes. As shown in Additional file 1: Figure S1 (c) and (d), whole-genome sequences of human and mouse exhibit lower *μ*/*ν* ratios (between 0.01 and 0.1) than protein-coding regions. Furthermore, the *μ*/*ν* ratio is significantly heterogenous within and across genomes; different chromosomes of human and mouse show *μ*/*ν* ratios varying from 0.001 to 0.1, see Additional file 1: Figure S2. Therefore, the *μ*/*ν* ratios we selected for the simulations in Fig. 5 should be typical and realistic for mammalian genomes.

### Benchmark on simulations against Lastz net alignment and BLAST RBH

Figure 5 displays the outcome of our simulation. For each *μ*/*ν* ratio, we evolve the diverged lineages until they become extremely distant to each other, and we plot in the figure the precision and recall of our method against the evolutionary distance between their genomes. The x-axis (“primary orthologous residue”) of the figure shows the proportion of genome bases that are covered by conserved primary orthologs, an indicator of the evolutionary distance between the diverged lineages; as the distance between genomes of these lineages increases, this proportion decays to zero. As a reference for the relevant range, we draw vertical dashed lines in all subfigures indicating the evolutionary distances between the human-chimpanzee (~ 93%) and human-mouse (~ 35%) genomes. In contrast to synthetic sequences evolved in silico, for natural genomes we have no record of which sequences are in fact primary orthologous; for natural genomes, we measure their evolutionary distances by the proportion of non-nested exact matches longer than 20 nucleotide bases.

To assess the performance of our method, we also apply two traditional alignment tools to the lineages obtained by our simulation: Lastz net alignment [54, 55, 56] and BLAST Reciprocal Best Hits (BLAST RBHs), both of which are expected to predict primary orthologs on the nucleotide level. We plot precision and recall of these two alignments within the same figure for comparison. It turns out that non-nested exact matches exhibit lower recall than Lastz net alignment within most regions of the figure: under *neutral* evolution, negative selection plays no role and exact matches are readily disrupted by point mutations, leading to misidentification of primary orthologs as in Fig. 3 (b). In contrast, alignment methods tolerate inexact matches and are less sensitive to those mutations; consequently, non-nested exact matches extract fewer primary orthologs than Lastz net alignment. On the other hand, non-nested exact matches exhibit higher precision than both alignment methods when the *μ*/*ν* ratio is not too high (as shown in Fig. 5 (a), (c) and (e)); at the same time, they also identify considerable amount of primary orthologs (as in Fig. 5 (b), (d) and (f)) thus the method is quite effective. Only when the *μ*/*ν* ratio is extremely high as in Fig. 5 (g) and (h), does the precision and recall of non-nested exact matches both fall below those of the alignment methods; our method based on non-nested exact matches becomes less effective in the limit of large *μ*/*ν*.

One way to improve the performance of our method is to account for point mutations under *neutral* evolution by incorporating inexact matches. In Fig. 5 we additionally employ *non-nested EDMs* to predict primary orthologs: each EDM contains a series of exact matches; lengths of contiguous mismatches between neighboring exact matches do not exceed *d* nucleotide bases (see section 2.2 for details). Non-nested EDMs perform better than non-nested exact matches in both precision and recall. As shown in Fig. 5, for most parameter values non-nested EDMs exhibit the highest precision among all methods; except when the diverged genomes evolve distantly (primary orthologous residue < 20%) with an extremely high *μ*/*ν* ratio (for example *μ*/*ν* = 1), Lastz net alignment exhibits higher precision (Fig. 5 (g)). With such a high *μ*/*ν* ratio, we also observe large standard deviations within the precision and recall of the alignment methods (see Additional file 1: Figure S3 and S4), implying that such a *μ*/*ν* ratio is probably too high to be realistic; in Additional file 1: Figure S1 (c)-(d) and Additional file 1: Figure S2, we observe that the *μ*/*ν* ratio for all sequences of natural genomes (both protein-coding and non-protein-coding) are typically lower than 1.

With varying evolutionary distance between the compared genomes, the precision and recall of Lastz net alignment vary non-monotonically. When the genomes are closely related (primary orthologous residue > 50%) in the presence of many duplications that haven’t yet diverged, Lastz net alignment often doesn't discriminate between parent and daughter copies of these duplications. Therefore it exhibits lower precision and lower recall than both non-nested exact matches and non-nested EDMs. For distantly-related genomes, Lastz net alignment exhibits higher recall that may be attributable to long-range genomic context and to the heuristic chaining algorithm employed by alignment. In contrast, extension is limited within our simple and naive EDM algorithm; many orthologs recovered in syntenic alignment chains, especially short ones, are lost to non-nested EDMs. Therefore, our method is more applicable to closely related genomes than to distantly related genomes.

Finally, non-nested EDMs yield greater precision and recall than BLAST RBHs. When the *μ*/*ν* ratio is low, BLAST recovers many pairs of matches between the compared genomes, but very few of these matches constitute *reciprocal* best hits; therefore BLAST RBHs exhibit very low recall. When *μ*/*ν* ratio is high, BLAST RBHs become unstable, exhibiting strong deviations in both precision and recall (see Additional file 1: Figure S4).

In our segmental duplication simulation, duplicated sequence replaces a sequence of the same length at a random position in the same genome so that genome size remains constant. However, as observed by an anonymous reviewer, in natural genomes segmental duplication usually involves insertion into a genome, increasing its length. The dynamics we implemented is equivalent to a duplication followed by a deletion of the same length at the insertion site. Limited evidence we have so far acquired suggests that qualitatively, duplication-insertion behaves like duplication-substitution, which lightens the computation burden for both numerical evolution and subsequent analysis. In Additional file 1: Figure S6, we indicate a preliminary study with the duplication-insertion model. It turns out that the result in Additional file 1: Figure S6 is qualitatively consistent with the result in Fig. 5: non-nested EDM has a higher precision, but lower recall, than Lastz net alignment for most parameter values.

### Natural genomes: performance of our method on the gene level

In this section, we extend inference of primary orthologs from the nucleotide to the gene level: we harness the method proposed above to identify primary orthologous genes between a pair of genomes. Genes are generally much longer than single maximal matches; a pair of homologous genes may encompass many maximal matches, some non-nested and others nested. We anticipate that *each pair of primary orthologous genes between two genomes shares more non-nested maximal matches with each other, than either of them shares with any other genes in the other genome*, leading naturally to a version of ***reciprocal best hit*** (RBH). In practice, by accumulating the number of non-nested maximal matches (weighted by length in bases) shared between each pair of genes from two genomes as a “hit score”, we obtain the RBHs of genes (see Methods for details) as candidates for primary orthologous gene pairs. To distinguish them from the conventional BLAST RBHs, which are determined by alignment score, we call RBHs determined by non-nested maximal matches ***non-nested RBHs***. In this paper, we determine non-nested RBHs of genes by non-nested exact matches no shorter than 20 nucleotide bases; application of non-nested EDMs to natural genomes exceeds the scope of this paper.

### Benchmark on natural genomes against Ensembl Compara and BLAST RBH

To assess the effectiveness of non-nested RBHs, we compare them to the orthologous genes annotated by Ensembl Compara, and to the Reciprocal Best Hits of genes determined by BLAST (i.e., BLAST RBHs) [39, 40, 41, 42, 43, 44]. Comparison of non-nested RBHs with the BLAST RBHs is straightforward, since both yield one-to-one related pairs of genes that are expected to be primary orthologs. However, Ensembl Compara does not identify primary orthologs; instead it classifies all orthologs identified from gene trees as *one-to-one*, *one-to-many* and *many-to-many* [8, 57]. Comparing to our classification of homologs in Table 1, it is evident that one-to-one orthologs of Ensembl are all primary orthologs; but one-to-many and many-to-many orthologs are by definition co-orthologs, and require further elucidation. Since Ensembl does not provide sufficient information to distinguish parent and daughter copies of duplications from each other; it is not possible following *solely* the Ensembl annotations to distill primary orthologs from all pairs of co-orthologs. Nevertheless, we observe that orthologs between a pair of genomes naturally cluster into *complete bipartite graphs*, which enables us to compare the primary orthologs yielded by Ensembl with those yielded by the RBH approaches.

### Complete bipartite graphs of orthologs (CBG)

**Fig. 6:**
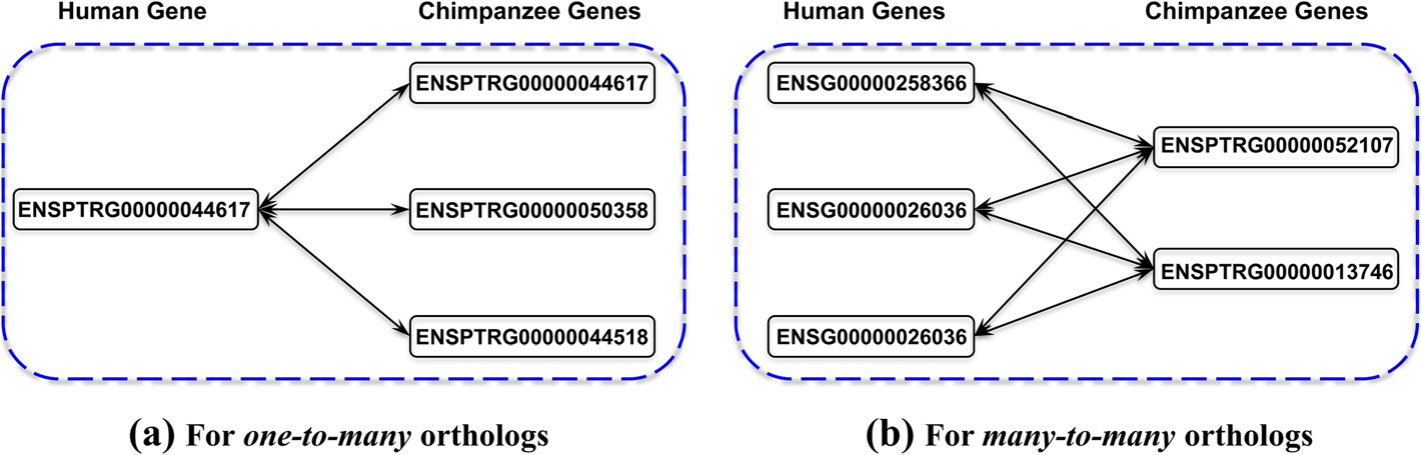
Complete bipartite graphs of orthologs between the human and chimpanzee genomes: (**a**) for *one-to-many* orthologs; (**b**) for *many-to-many* orthologs. In each subfigure, the complete bipartite graph is indicated by the dashed rectangle, and genes are named by their Ensembl stable ids [58]

We observed that orthologs between a pair of genomes naturally cluster into *complete bipartite graphs* (CBG as shown in Fig. 6), which satisfy the two conditions:

(1) Within each CBG, every gene from one genome is orthologous to every gene from the other genome: orthology only applies to gene pairs from different genomes; genes from the same genome are all paralogous to one another; and.

(2) For each gene in a given CBG, its orthologous genes in the other genome are *all* members of the same CBG, so that the bipartite graph is not only “complete,” but also “maximal.”

This “CBG of orthologs” should not be confused with the “clique of orthologs” discussed elsewhere [9, 59]; the notion of “clique of orthologs” applies to multiple genomes, whereas CBG solely reflects orthology between a *pair* of genomes. By definition, each CBG contains at most one pair of primary orthologs; and in the event of gene loss, for example, no primary orthologs at all. Therefore the total number of CBGs is presumably no smaller than the total number of primary orthologs. This observation enables us to estimate the maximal number of primary orthologs predicted by Ensembl, and to evaluate whether the RBH and Ensembl CBG classifications are consistent.

### Consistency between RBHs and Ensembl CBGs

**Fig. 7:**
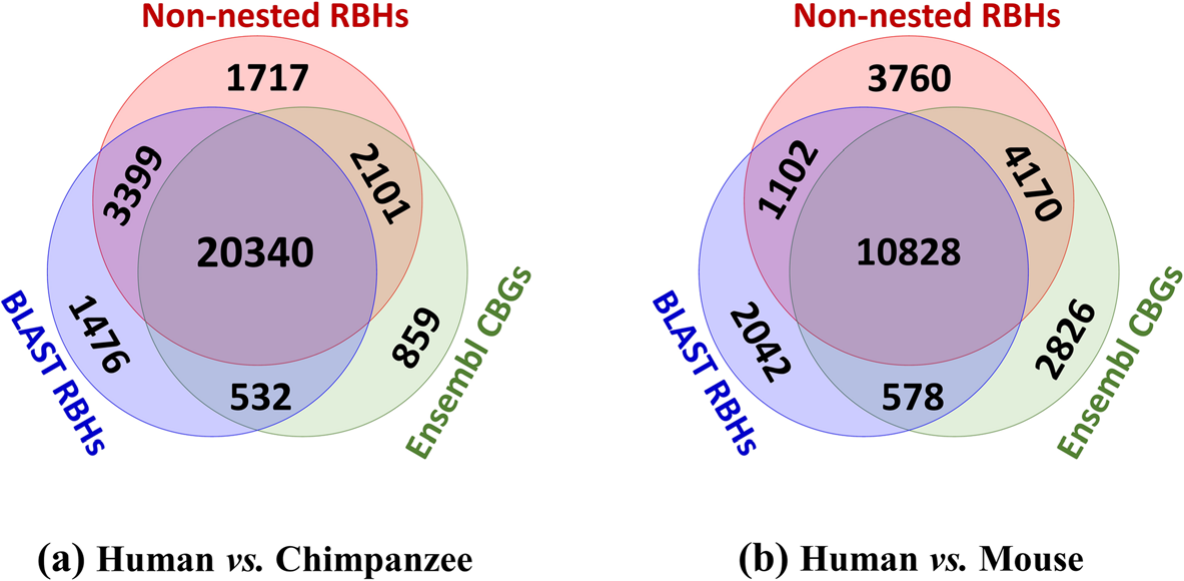
Venn diagrams among three sets of orthologous genes: non-nested RBHs, BLAST RBHs and Ensembl CBGs, between the genomes of (**a**) human-chimpanzee and (**b**) human-mouse. Non-nested RBHs in this figure are determined by non-nested exact matches no shorter than 20 bases; numbers in the figure indicate numbers of gene pairs, unweighted by their lengths in bases

Figure 7 shows Venn diagrams among three sets of orthologous genes: non-nested RBHs, BLAST RBHs, and Ensembl CBGs of orthologs; each set is represented by a circle. Overlaps among different circles indicate subsets of orthologous genes shared among different sets. Numbers in Fig. 7 indicate counts of orthologous gene pairs without weighting by length. Note that each Ensembl CBG contains multiple pairs of orthologous genes, among which by definition only one pair is primary orthologs. The information provided by Ensembl does not enable identification of this pair, complicating any comparison between the set of primary orthologs yielded by Ensembl CBGs with those yielded by the RBH approaches. To compare non-nested RBHs and Ensembl CBGs, we adopt the following strategy: when there is one, and only one, pair of orthologous genes within a given Ensembl CBG that is identified by our method as a non-nested RBH, the outcome is designated as “consistent” and we increment by one the number of gene pairs common to Ensembl CBGs and non-nested RBHs. Venn diagrams of Ensembl CBGs and BLAST RBHs are computed similarly.

Table 3 displays for Fig. 7 the precision and recall of our non-nested RBHs benchmarked by both BLAST RBHs and Ensembl CBGs. It demonstrates that among all non-nested RBHs between human and chimpanzee genomes, up to 94% are validated by either BLAST RBHs or Ensembl CBGs, and nearly 74% are validated by both of them; for human-mouse, the corresponding proportions are 81 and 55%. Having been validated by at least one other method, these non-nested RBHs can be regarded with high confidence as primary orthologs. On the other hand, non-nested RBHs also recover more than 90% of the primary orthologs between human and chimpanzee identified by BLAST RBHs or Ensembl CBGs, and over 80% of those for human-mouse. Therefore, combined with an RBH approach, our method predicts primary orthologous genes with both high precision and high recall.

**Table 3:** Statistics for Fig. 7 exhibiting the precision and recall of non-nested RBHs benchmarked by both BLAST RBHs and Ensembl CBGs. Numbers of RBHs (or CBGs) shared by the corresponding sets are indicated in parentheses

Genome Pairs	Human-Chimpanzee	Human-Mouse
Total number of non-nested RBHs	27,557	19,860
Precision 1: *Non-nested RBHs validated by either BLAST* *RBHs or Ensembl CBGs*	93.8% (25840)	81.1% (16100)
Precision 2: *Non-nested RBHs validated by both BLAST* *RBHs and Ensembl CBGs*	73.8% (20340)	54.5% (10828)
Recall 1: *Ensembl CBGs recovered by non-nested RBHs*	94.2% (22441)	81.5% (14998)
Recall 2: *BLAST RBHs recovered by non-nested RBHs*	92.2% (23739)	82.0% (11930)

Non-nested RBHs also yield novel putative gene orthology that other methods fail to recover. For example, among all non-nested RBHs between human and chimpanzee genomes, 999 of them consist of human and chimpanzee genes that do not appear at all within BLAST RBHs and Ensembl annotations for homology: both BLAST RBHs and Ensembl Compara fail to predict homology for these genes, and the orthology predicted by non-nested RBHs for these genes contradicts neither BLAST RBHs nor Ensembl Compara. We anticipate many of these non-nested RBHs represent novel predictions of gene orthology that will stand the test of time. On checking the annotations for these 999 genes, we found 935 (more than 93%) of them are as yet “uncharacterized” or annotated as “novel” genes. The remaining 64 have already been annotated; most of them consist of human and chimpanzee genes that have very similar, or even identical annotations (see Additional file 1: Supplementary material 2 for a list of these genes with their annotations). To our knowledge, genes are usually annotated based on homology. The novel putative orthology discovered here for these uncharacterized genes should facilitate their proper annotation.

Both BLAST RBHs and our method are based on sequence similarity; in contrast, the pipeline of Ensembl Compara involves reconciliation and is not merely similarity-based. Taking Ensembl CBGs as a common benchmark, in Table 4 we compare the performance of our method with BLAST RBHs. It can be seen that for both human-chimpanzee and human-mouse, our non-nested RBHs predict more pairs of primary orthologs and recover more Ensembl CBGs than do BLAST RBHs.

**Table 4:** Statistic for Fig. 7, exhibiting the precision and recall of non-nested RBHs and BLAST RBHs, benchmarked by Ensembl CBGs. Numbers in the brackets indicate the numbers of RBHs (or CBGs) shared between the corresponding sets

Genome Pairs	Human-Chimpanzee	Human-Mouse
Total number of non-nested RBHs	27,557	19,860
Precision: *Non-nested RBHs validated by Ensembl CBGs*	81.4% (22441)	75.5% (14998)
Recall: *Ensembl CBGs recovered by non-nested RBHs*	94.2% (22441)	81.5% (14998)
Total number of BLAST RBHs	25,747	14,550
Precision: *BLAST RBHs validated by Ensembl CBGs*	81.1% (20872)	78.4% (11406)
Recall: *Ensembl CBGs recovered by BLAST RBHs*	87.6% (20872)	62.0% (11406)

### Examination of additional species pairs

The above observation extends to more species. In Additional file 1: Figure S7, we implement the same methods as in Fig. 7 on seven pairs of genomes over a range of evolutionary distances, including human-gorilla, human-cat, human-chicken, human-lizard, human-frog, human-fish and human-drosophila; the statistic of Table 4 is shown in Additional file 1: Table S1. When genomes are more distantly related, accumulated sequence variation within the genomes decreases sequence similarity, so that methods based *solely* on sequence similarity gradually lose their effectiveness. In Additional file 1: Figure S7 and Additional file 1: Table S1, we observe lower precision and recall than in Tables 3 and 4 both for BLAST RBHs and for our method. However, within between human and another vertebrates, the precision of our method benchmarked by Ensembl CBGs still exceeds 60%; the proportion of non-nested RBHs validated either by BLAST RBHs or by Ensembl CBGs exceeds 80% for human-bird, human-reptile and human-amphibians, and approaches 70% for human-fish. On the other hand, as in Table 4, our method always recovers more primary orthologs that are consistent with Ensembl CBGs than does BLAST RBH; in Additional file 1: Table S1 our method exhibits superior precision and recall. Therefore, in many cases our method can replace BLAST RBHs and predict primary orthologous genes effectively. These observations support our proposal on the relationship between non-nested maximal matches and primary orthologs.

### Indispensability of non-nested maximal matches

Finally, reciprocal best hit (RBH) is an independent method that infers one-to-one ortholog relationships [60]; its effectiveness has been investigated in previous literature [42]. A natural question is whether the performance of our method exhibited above can be attributed mainly to the *non-nested maximal matches* that determine the RBHs, or to RBH alone. To ascertain the role of non-nested maximal matches, we extracted the mmRBHs of genes determined by *all* exact matches (both non-nested and nested), and evaluated their consistency with Ensembl CBGs and BLAST RBHs; see Additional file 1: Table S2. The performance of such a procedure depends critically on the minimal length of exact matches; for human-chimpanzee, it shows even higher precision than exhibited in Table 3; however it still exhibits lower recall. Our explanation for this is that between very closely related genomes such as human and chimpanzee, non-nested maximal matches overwhelmingly dominate nested ones; especially when the maximal matches are long, an overwhelming majority of hits (i.e., matches) on genes between these two genomes are non-nested (see parameter *P* in Additional file 1: Table S2). In this case, the mmRBHs determined by all exact matches on the one hand and solely by non-nested exact matches on the are nearly equivalent. But for human-mouse, non-nested exact matches dominate less strongly than for human-chimpanzee. As a result, for human-mouse we obtained many fewer (less than 1/7) of the non-nested RBHs; recall is greatly reduced. This result is consistent with [60]. Therefore, the high performance of our method in Fig. 7 and Table 3 can’t be attributed solely to RBH: ***the role of non-nested maximal matches is essential****.*

## Discussion

### Similarity-based methods and tree-based methods

Ortholog identification methods are customarily divided into two categories: similarity-based methods and tree-based methods. Similarity-based methods (also known as “graph-based” methods) evaluate one pair of genomes at a time; inference relies on pairwise all-versus-all sequence comparison. Tree-based methods deal with multiple genomes at a time; they infer orthology and paralogy from phylogenetic trees, and usually need to reconcile gene trees with species trees [9]. Compared to similarity-based methods, tree-based methods are more computationally intensive for large datasets, and are more difficult to automate.

A further strength of similarity-based methods over pure tree-based methods is that they predict primary orthologs. The orthology pipeline of Ensembl is primarily tree-based; thus it does not identify primary orthologs. In this paper, through Ensembl CBGs we could infer only the maximal *numbers* of primary orthologs, but not exactly which pairs of genes are primary orthologs. On the other hand, because of gene loss the number of conserved primary orthologs could fall below the number of Ensembl CBGs, especially for distantly related species. With Ensembl CBGs as benchmark, the recall of our method (and also that of BLAST RBHs) on primary orthologs as reported in in Table 3, Table 4, and especially in Additional file 1: Table S1, is very likely to be an underestimate.

The method proposed in this paper is also similarity-based. Technically it infers primary orthologs as those sequences or genes with the highest similarity; the novelty is that we consider local genomic contexts when measuring the similarity. We attribute the effectiveness of our method to either or both of the following two hypotheses: (1) primary/positional orthologs tend to conserve their ancestral positions in the genomes [19]; and (2) primary orthologous regions tend to undergo lower variation rate than non-orthologous regions [9]. Among duplicates that have diverged under asymmetrical evolution, it is possible to discriminate primary orthologs from other homologs merely by comparing the sequences within the duplicated regions; some alignment methods discriminate primary orthologs on this basis. However, (i) when asymmetrical evolution does *not* apply; or (ii) between recently emerged paralogs that haven’t diverged yet, sequence similarity within the duplicated regions is not always sufficient to distinguish primary and non-primary orthologs, and conserved genomic contexts of primary orthologs favor the effectiveness of our method. As in the numerical simulations shown in Fig. 5, when the genome evolution is neutral and asymmetrical evolution plays no role, our method remains effective, although it shows lower recall than Lastz net alignment. On the other hand, in the event of translocation or gene loss the positional ortholog is disrupted; based on the hypothesis of asymmetrical evolution, our method extracts non-positional orthologs that have the highest sequence similarity as primary orthologs. Therefore, as opposed to the term “positional ortholog,” we suggest the term “primary ortholog” is more suitable to describe the orthologs extracted by our method.

### Sequence orthology and gene orthology

**Fig. 8:**
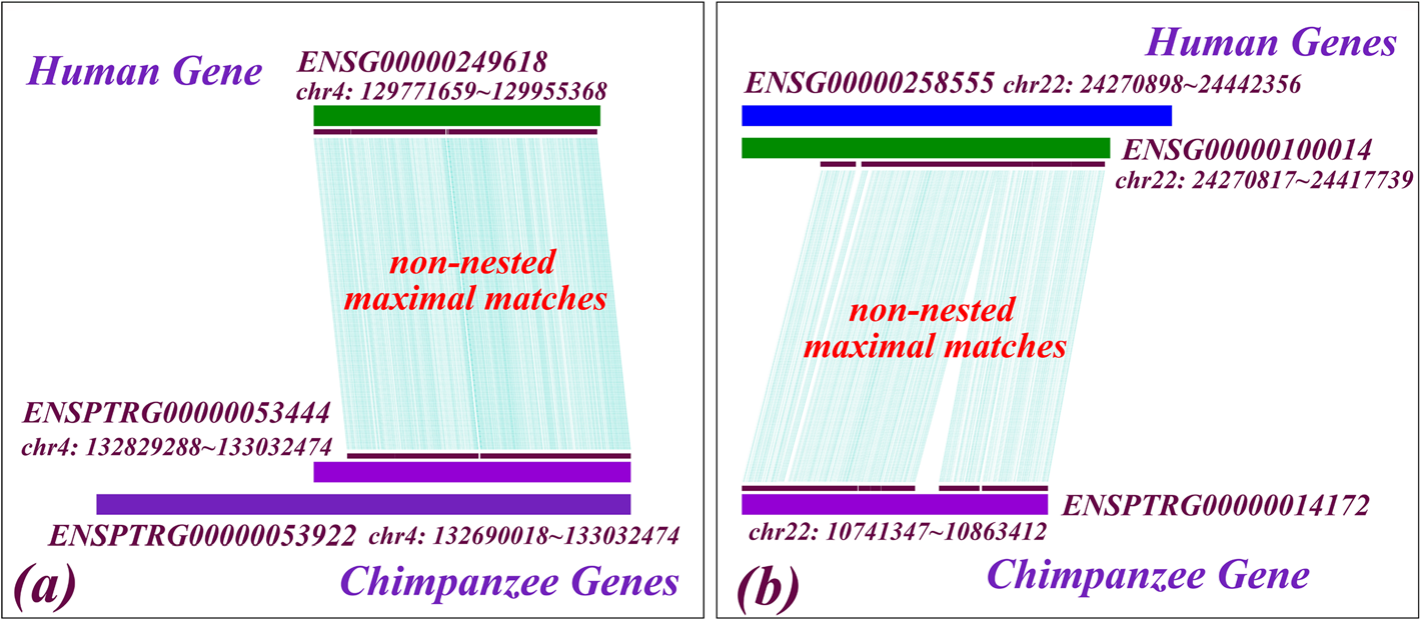
Overlap between genomic regions of different genes may complicate the inference of gene orthology. In each subfigure, genomic regions of two genes in one genome overlap strongly with each other, and all non-nested maximal matches shared with a certain gene in the other genome are found within the overlapping region. Without inferring the evolutionary histories of these genes via outgroup genes, automated sequence comparison alone is not able to distinguish those pairs of genes that are actually primary orthologous

Essentially, orthology reflects the evolutionary history of genomic regions (i.e., sequences), but not necessarily gene function; gene orthology is usually derived from sequence orthology. However, similarity-based methods do not directly infer the evolutionary history; sometimes they may yield the right sequence orthology but not the right gene orthology. For example, overlap between different genes within the same genome may yield ambiguity when inferring gene orthology via sequence orthology between a pair of genomes. Figure 8 shows two examples; in each example, large parts of two genes within one genome overlap with each other, and all non-nested maximal matches shared with a certain gene of another genome appear within the overlapping region, suggesting that it is actually this overlapping region that is orthologous to the gene in the second genome against which both genes in the first genome exhibit the same hit score. In such a case, our method correctly infers the sequence orthology between the corresponding genomic regions, but can’t determine which of these gene pairs holds the “true” gene orthology; the latter can only be inferred by a tree-based method through the evolutionary history via outgroup genes. Such examples are ubiquitous in natural genomes; we found hundreds of them between human and chimpanzee genomes. Such a discrepancy is presumably beyond the scope of a sequence-based method of ortholog identification.

The well-known “*ortholog conjecture*” asserts that it is usually the parent copy of a duplication that retains the function of the ancestor, whereas the daughter copy tends to accumulate variations and develop a new function [9]. It is beyond the scope of this paper to discuss the “mechanism” behind such a phenomenon, but to the extent that the primary ortholog retains its position within the linear structure of the genome, it presumably maintains its (time-dependent) coordinates in three dimensional space within the nucleus, perhaps in the neighborhood of a nuclear envelope, or of a “transcription factory,” to name a couple of quite arbitrary but plausible alternatives. In other words, the local environment of the genome within the cell is not translationally invariant, although when inspecting a genome sequence it is easy to lose sight of that. Thus, a “mechanism” could be readily at hand.

A primary aim in this paper was to develop methods to assess sequence- as opposed to gene- orthology, for application to non-coding as well as to coding sequences. Because of the redundancy of the genetic code, protein homology can be detected even when genomes have diverged too far for sequence homology to be readily detected. Thus, for all but closely related species, comparisons are often performed on the level of protein sequence, rather than nucleotide sequence.

If the aim were instead to specifically assess orthology of protein-coding genes, it would be helpful if measures of protein sequence homology, as opposed to nucleotide sequence homology, could be incorporated into the methodology we have proposed. Unfortunately, once alignments are restricted to protein-coding regions so that measures of protein sequence homology can be readily applied, it becomes far from obvious whether the same notions of nesting will be effective.

On the one hand, it could be that the method sometimes proves effective when directly applied to translated coding sequence, although certain associated technical challenges could arise. On the other hand, as an anonymous reviewer proposes, it is plausible that for homologous protein sequences, homology of their cis-regulatory non-coding sequences could be harnessed to assess orthology by determining whether or not the non-coding homology remains associated with the coding homology, or decouples from it. This idea could be viewed as a higher-level abstraction of the concept of nesting, and seems to us worthwhile to pursue in the future.

### Synteny, genomic context and context-based alignment

The term “synteny” is widely invoked to refer to homologous regions with *conserved gene order*, although this colloquial application is not consistent with the original definition of the term [61, 62]. Synteny information represents a form of genomic context over long regions of genomes; it has been widely used for predicting or refining orthologous relationships. In previous literature, synteny is commonly taken as synonymous with orthology, and “genomic context” generally refers to “synteny information.” It has been observed that synteny-based inference of orthology yields high concordance with sequence-based inference of orthology [63]. For example, Blastz/Lastz alignment exploits synteny information for chaining; the incorporation of synteny information enables it to predict primary orthologs [55].

In contrast, the work in this paper for the first time extends the idea of context-based ortholog identification from long-range contexts to short-range ones—as short as single maximal matches—that we demonstrate provide an effective means to identify orthologs within whole genomes. The method of non-nested maximal matches proposed in this paper relies on local nesting of sequences; synteny information plays no direct role. Compared to synteny-based methods, non-nested maximal matches show higher precision but lower recall in ortholog identification under a *neutral* evolution model, as we have observed in Fig. 5. In particular, such a method tends to miss orthologs that are relatively short (for example, shorter than 30 bases), because short matches are likely to be nested in other matches. In contrast, synteny-based methods such as Lastz alignment recover short orthologs through chain extension: a syntenic chain of HSPs (high-scoring segment pairs) can encompass many short orthologs. However, in natural genomes non-nested maximal matches coincide well with Lastz net alignment among relatively long sequences.

When the compared genomes are repeat-masked as a prerequisite to Lastz alignment, Lastz net alignment recovers fewer orthologs within the repeat-masked regions than are recovered by non-nested maximal matches, which can be obtained without repeat-masking. Table 5 shows an example in natural genomes: in regions of whole-genomes, non-nested exact matches recover a large majority (> 95%) of all exact matches longer than 30 bases of a Lastz net alignment, suggesting that although non-nested maximal matches are not defined in terms of synteny, many of the in-synteny sequences longer than 30 bases turn out to be non-nested. On the other hand, Lastz net alignment yields lower coverage of non-nested exact matches: certain of the elements contributing to non-nested exact matches are lost to the Lastz net alignment. These elements turn out to appear primarily in *repeat-masked regions* of the genomes; in genome regions that are not repeat-masked, non-nested maximal matches and Lastz net alignment are quite consistent with one another (mutual coverage > 90%). Therefore, our method can overcome certain adverse effects of repeat-masking on the Lastz alignment by better identifying orthologs within the repeat-masked regions of genomes.

**Table 5:** Comparison between (i) non-nested exact matches (*nnem*) and (ii) maximal matches from Lastz net alignment (*net*). Matches compared in this table exceed 30 nucleotide bases in length. Percentages show the conditional probabilities that a maximal match is in one set given that it is in the other. As a prerequisite for Lastz net alignment, the whole genomes were preprocessed by repeat-masker; NRM regions are those parts of the genome that repeat-masker did not mask

Genome Pairs	Conditional Probabilities	Forward Strand		Reverse Strand	
		Whole Genome	NRM Regions	Whole Genome	NRM Regions
Human vs. Chimpanzee	*P*(*net | nnem*)	87.9%	97.8%	46.9%	88.9%
	*P*(*nnem | net*)	96.4%	98.7%	91.5%	94.0%
Human vs. Mouse	*P*(*net | nnem*)	12.9%	92.0%	11.2%	90.5%
	*P*(*nnem | net*)	97.1%	97.7%	96.6%	97.2%

### Intersection and alignment

Genome alignment arranges sequences to identify regions of similarity that may have arisen from evolutionary relationships [64]. A typical genome alignment consists of two phases: (1) a seed/search phase involving an all-against-all search for highly similar or identical sequences between the compared genomes; and (2) a subsequent mapping/clustering phase that concatenates these sequences. Intersection, as defined in Methods, is one option for the first phase of alignment; alignments that perform intersection as a first step include, for example, *MUMmer* [32, 33, 34, 35]. An intersection exhaustively recovers all sequences shared by the compared genomes, without any filtering or assembling. The term “intersection” was applied in [65]; elsewhere, e.g. in [46], intersection is referred to as “alignment” although according to our use of the term it is only a preliminary stage thereof and would require in addition some form of mapping or clustering to qualify as an alignment.

For many purposes including our own the distinction between intersection and alignment is important. Our method based on non-nested maximal matches, although it can be performed on an alignment (see section 3.2.4 in [36]), does not require a comprehensive alignment to identify and classify orthologs; intersection is sufficient. In contrast to alignment-based methods that are algorithmically defined, intersection-based methods like ours exhibit certain virtues:

1. Intersection involves fewer parameters; the objects computed can be precisely defined, reducing the potential for artifacts arising from choice of parameters or other unexpected outcomes of the alignment algorithm.
2. Intersection can be implemented on a wider range of sequences: Blastz/Lastz alignment requires prior repeat-masking of the sequences to be aligned [38]; consequently, it fails to identify many orthologs in repeat-masked regions of the genome (see Table 5). In contrast, intersection can be implemented as in this paper directly on whole genomes, with no repeat-masking, so that our method detects more orthologs than Lastz within the repeat-masked regions of genomes.
3. With existing computational technology, intersection can be more efficiently performed than many widely used alignment tools such as Blastz/Lastz; for a given pair of sequences, intersection usually consumes much less computational time than alignment, particularly for whole-genome comparisons.

For example, with a single core of Intel Xeon E5-2680v3 processor at 2.50 GHz on our high performance computation cluster, an intersection with *SEQANALYSIS* together with a full classification of “super, nested local and non-nested local” occurrences (see Methods and [29] for details) between a single pair of chromosomes of human and chimpanzee, neither of which were repeat-masked, requires around 20 min of computation time. In contrast, a raw alignment with Lastz for the same pair of chromosomes takes more than 4 h, not including the time required for the essential pre-alignment process of repeat- masking on those chromosomes. When comparing whole genomes, an intersection with *SEQANALYSIS* between 3.1 GB of human sequence and 3.2 GB of chimpanzee sequence requires less than 24 h, whereas the corresponding Lastz raw alignment can take more than 720 h of computation time with a single core of CPU on our cluster. Recently, a new algorithm was developed to perform the context-sensitive maxmer classification in linear time [66] that may further improve the computational efficiency of our intersections.

(4) When the genome database is updated, intersection-based methods can be scalable and extensible. When annotations of genes, but not genomic sequences, are revised, whole-genome intersections and classifications of non-nested/nested maximal matches remain valid; we need only recalculate the hit scores on those genes whose annotations have been revised, together with the corresponding mmRBHs. On the other hand, when the intersection needs to be extended, for example, from maxmers longer than 30 bases to maxmers longer than 20 bases, we need only classify in addition the non-nested and nested maximal matches with lengths between 20 and 30 bases; non-nested and nested maximal matches longer than 30 bases remain valid.

### EDMs

In Results, we applied non-nested EDMs in our simulation to account for point mutations under a neutral evolution model. EDMs require identical separations between matches in different genomes; in contrast, chaining/clustering in an alignment typically admits variable separations. The constraint on EDMs has on the one hand enabled us to extract them easily from an intersection, but on the other hand does not account for indels (short insertions and deletions). In natural genomes, indels usually occur at a lower rate than point mutations and segmental duplications. For example, within Lastz raw self-alignment of human chromosome 1, the frequency of indels averaged over all alignment blocks is around 2%, whereas the average frequency of point mutations is around 32%; within Lastz net alignment between human chromosome 1 and chimpanzee chromosome 1, the corresponding frequencies are 0.5 and 23% respectively. Although there might be some ascertainment bias, the frequency of indels is lower by at least an order of magnitude than the frequency of point mutations. Therefore, in our numerical simulation, we study the impact of segmental duplication and point mutation, but ignore the impact of indels.

One way to account for indels could be to invoke a clustering algorithm, such as *gaps* or *mgaps* of *MUMmer* [32, 33, 34, 35] on exact matches extracted by an intersection. A clustering algorithm will account for longer stretches of genomic context than our method; it creates a local alignment, within which non-nested/nested maximal matches can be discriminated. In [36], we have shown the effectiveness of such “non-nested/nested CMRs” derived from an alignment between single chromosomes of human and chimpanzee. However, a practical method to obtain the nesting structure genome-wide among those clusters as efficiently as we did here for exact matches and EDMs remains unknown to us.

In this paper, EDMs were applied only to numerical simulations within a neutral evolution model; presumably because of selection, non-nested exact matches alone work well for natural genomes. Non-nested exact matches can be efficiently computed on whole-genome sequences through an intersection; the effectiveness of mmRBHs determined by non-nested exact matches was demonstrated in the Results section. In contrast, with existing algorithms, genome-wide identification of non-nested EDMs is more computationally intensive. Although we expect non-nested EDMs to perform better than non-nested exact matches on natural genomes, we compromise between performance and efficiency in this paper. A more efficient algorithm to extract non-nested EDMs from whole-genome sequences remains to be developed, and the application of non-nested EDMs to natural genomes awaits further investigation.

## Conclusions

The customary definition of “co-ortholog” notwithstanding, there is a certain consensus on the practical utility of a potential “one ortholog one organism” relationship. Among a group of co-orthologous genes, we *assume* that there is one and only one “most orthologous” gene pair that is more likely to play equivalent roles within both genomes than are other gene pairs. It is an empirical matter as to whether this assumption is borne out in practice. Following the definitions developed in previous literature [9, 19, 20], we choose the notion of “primary ortholog” to describe such “most orthologous” relationship among genes or sequences. Primary orthologs are assumed to retain their common ancestor’s positions in the genome, and their evolution is presumably constrained chiefly by negative selection; in contrast, the evolution of secondary orthologs evidently more resembles that of paralogs [36]. These observations are consistent with those of Arndt and coworkers [67].

Primary orthologs can be identified through a similarity-based method in combination with information contained in the genomic context [19]. As we have proposed in this paper,

- The local structure of non-nested/nested maximal matches can efficiently discriminate primary orthologs from other homologs on the nucleotide level. With a simple and naive algorithm, non-nested maximal matches extract primary orthologs with higher precision than an alignment method, even under neutral evolution alone.
- In natural genomes, above a certain minimum length, non-nested maximal matches recover most of those sequences obtained by Lastz net alignment; this consistency suggests a potential relationship between short-range genomic context and long-range genomic contexts, such as synteny. In regions of genomes that would be masked by repeat-masker, non-nested maximal matches recover orthologs that are lost to Lastz net alignment, for which repeat-masking is a prerequisite.
- In combination with a reciprocal best-hit approach, non-nested maximal matches elucidate primary orthologs not only for sequences but also for genes. Reciprocal best hits of genes determined by non-nested maximal matches (non-nested RBHs) recover primary orthologous genes with higher precision and higher recall than BLAST RBHs; the inferred primary orthologous genes are consistent with the complete bipartite graphs (CBG) of orthologous genes derived from the annotations of Ensembl Compara.
- Non-nested maximal matches can be retrieved by either a genomic intersection or a genomic alignment [36]; the intersection-based calculation involves fewer parameters so that the computed objects are simple to elucidate and can be efficiently implemented.

Inasmuch as relatively short contigs alone should suffice for the intersection-based computations reported here, the prospect arises that for purposes of identifying and inferring the evolutionary history of orthologs genome-wide, it may eventually be possible to bypass or significantly abridge the process of genome assembly.

## Supplementary information

**Additional file 1: Figure S1.** Match length distributions (MLDs) exhibited by histograms of maxmers for real genome sequences, as well as for synthetic sequences created by the model described in [46, 47]. Red curves in the figure exhibit the MLDs of the given real genome sequences, without any repeat-masking: (a) human whole-genome protein-coding sequences, (b) mouse whole-genome protein-coding sequences, (c) human whole-genome sequences, both protein-coding and non-protein-coding, and (d) mouse whole-genome sequences, both protein-coding and non-protein-coding. Other curves in the figure show MLDs for the synthetic sequences of the same length and the same maximal duplication length as the corresponding real genome sequence; different synthetic sequences are created with different *μ/ν* ratios. In each subfigure, by comparing the MLD of the real genome sequence to the MLDs of the synthetic sequences, we estimate the *μ/ν* ratio for the real genome sequence as following: (a) for human protein-coding genes, *μ/ν* ≈ 1; (b) for mouse protein-coding genes *μ/ν* ≈ 0.1; (c) and (d), for human and mouse whole-genome sequences, *μ/ν* is between 0.01 and 0.1. **Figure S2.** Match length distributions (MLDs) for different chromosomes of human and mouse, as well as for synthetic sequences of the same length and the same maximal duplication length, created by the model described in [46, 47] with different *μ/ν* ratios. As shown in the figure, the *μ/ν* ratio is significantly heterogenous within and across genomes; different chromosomes of human and mouse show very different *μ/ν* ratios, varying from 0.001 (as mouse chromosome Y in subfigure (i)) to over 0.1 (as human chromosome 5 in subfigure (a)). Therefore, the *μ/ν* ratios selected for the numerical simulations exhibited in Fig. 5 of the main text (*μ/ν* = 0.001, 0.01, 0.1 and 1) should be typical and realistic for mammalian genomes. **Figure S3.** Standard deviations indicated by error bars for the Lastz net alignment exhibited in Fig. 5 of the main text. **Figure S4.** Standard deviations indicated by error bars for the BLAST RBHs exhibited in Fig. 5 of the main text. **Figure S5.** Standard deviations indicated by error bars for the non-nested EDMs exhibited in Fig. 5 of the main text. **Figure S6.** Precision and recall of our method derived from numerical simulations, in which each segmental duplication is inserted into the genome rather than substitutes another sequence of the same length in the same genome. Due to computation burden, we only simulate diverged lineages whose similarity is above 20% in a single realization. The result is essentially consistent with that in Fig. 5 of the main text **Figure S7.** Venn diagrams among non-nested RBHs, BLAST RBHs and Ensembl CBGs between the genomes of human against four different species: (a) gorilla (*Gorilla gorilla*), (b) cat (*Felis catus*), (c) chicken (*Gallus gallus*), (d) anole lizard (*Anolis carolinensis*), (e) frog (*Xenopus tropicalis*), (f) zebrafish (*Danio rerio*), and (g) drosophila (*Drosophila melanogaster*). From (a) to (g), species are more and more distantly related to human; in this figure we use *MUMs* longer than 15 nucleotide bases to determine the non-nested RBHs. Estimated by the proportion of non-nested RBHs validated by *either* BLAST RBHs *or* Ensembl CBGs, the precision of our method in each subfigure is: (a) 94.5%, (b) 89.6%, (c) 93.8%, (d) 87.5%, (e) 84.7%, (f) 67.6% and (g) 27.8%. **Table S1.** Statistics for figure S7, exhibiting the precision and recall of non-nested RBHs and BLAST RBHs, benchmarked by Ensembl CBGs. Numbers in the brackets indicate the numbers of RBHs shared between the corresponding sets. **Table S2.** Performance of a “control experiment” in which mmRBHs are determined by all exact matches (both non-nested and nested). Parameters *Lmin* refers to the minimal length of exact matches, and *P* refers to the proportion of hits on genes contributed by non-nested exact matches over those contributed by all exact matches, both weighted by lengths in bases. Precisions and recalls are defined as same as those in Table 3 of the main text, except non-nested RBHs are substituted by mmRBHs determined by all exact matches: “Precision 1:” mmRBHs validated by either BLAST RBHs or Ensembl CBGs; “Precision 2:” mmRBHs validated by both BLAST RBHs and Ensembl CBGs; “Recall 1:” Ensembl CBGs recovered by mmRBHs; “Recall 2:” BLAST RBHs recovered by mmRBHs. Numbers in the brackets indicate the numbers of RBHs (or CBGs) shared by the corresponding sets. For human-chimpanzee, with large *Lmin*, non-nested exact matches overwhelmingly dominate nested ones in terms of hits on genes; mmRBHs determined by all exact matches and those solely by non-nested exact matches are nearly equivalent. For human-mouse, non-nested exact matches are not as dominant as for human-chimpanzee; therefore, for human-mouse, mmRBHs RBHs determined by all exact matches exhibit much lower precision and recall than those determined by non-nested exact matches. **Supplementary Material 1.** How to identify non-nested and nested maximal matches with *SEQANALYSIS*. **Supplementary Material 2.** Ensembl annotations for human and chimpanzee genes that constitute nonnested RBHs but are *not* annotated as orthologs by Ensembl Compara.

## Data Availability

For genome intersection we download whole-genome sequences of human [68], chimpanzee [69] and mouse [70] from the FTP server of Ensembl. In this paper, we use version 96 of the Ensembl databases; similar studies on version 76 and version 81 of Ensembl can be found in [71]. Gene annotations for all species can be obtained from the Ensembl core database. We extract the information we need from the corresponding tables of the MySQL server at Ensembl. Annotations of gene orthology can be obtained from the Ensembl Compara database; tables for the Compara database version 96 can be downloaded from [72]. We wrote simple code to read these tables and extract the entire list of Ensembl annotated orthologous genes. Alternatively, one can use the Ensembl APIs [73] to fetch the same data programmatically. For the comparison between intersection and alignment, we downloaded whole-genome pairwise alignment as compressed MAF files from the Ensembl FTP server: for the whole-genome alignment of human-chimpanzee [74], and for that of human-mouse [75]. Methods of extracting all exact matches from these alignments are described in [30] and [36]. Source code of our software package, *SEQANALYSIS*, can be obtained in the webpage of Physics and Biology Unit, Okinawa Institute of Science and Technology Graduate University [76]. A full instruction manual is also available with the package. All data sources for the calculations in this paper are publicly available.

## Abbreviations

BLAST RBHs: reciprocal best hits of genes determined by the alignment scores of BLAST
CMRs: contiguous matched runs
EDM: equidistant match
Ensembl CBGs: complete bipartite graphs of orthologs annotated by Ensembl Compara
HSPs: high-scoring segment pairs in a Lastz alignment
mmRBHs: reciprocal best hits of genes determined by a given group of maximal matches
MUMs: maximal unique matches
non-nested RBHs: reciprocal best hits of genes determined by non-nested maximal matches
