# Supplementary material for: Primary orthologs from local sequence context
Source: BMC Bioinformatics. 2020 Feb 6;21:48. doi: 10.1186/s12859-020-3384-2 (PMC7006074; doi:10.1186/s12859-020-3384-2)
Supplement: Supplementary file 1 — Additional file 1: Figure S1. Match length distributions (MLDs) exhibited by histograms of maxmers for real genome sequences, as well as for synthetic sequences created by the model described in [46, 47]. Red curves in the figure exhibit the MLDs of the given real genome sequences, without any repeat-masking: (a) human whole-genome protein-coding sequences, (b) mouse whole-genome protein-coding sequences, (c) human whole-genome sequences, both protein-coding and non-protein-coding, and (d) mouse whole-genome sequences, both protein-coding and non-protein-coding. Other curves in the figure show MLDs for the synthetic sequences of the same length and the same maximal duplication length as the corresponding real genome sequence; different synthetic sequences are created with different μ/ν ratios. In each subfigure, by comparing the MLD of the real genome sequence to the MLDs of the synthetic sequences, we estimate the μ/ν ratio for the real genome sequence as following: (a) for human protein-coding genes, μ/ν ≈ 1; (b) for mouse protein-coding genes μ/ν ≈ 0.1; (c) and (d), for human and mouse whole-genome sequences, μ/ν is between 0.01 and 0.1. Figure S2. Match length distributions (MLDs) for different chromosomes of human and mouse, as well as for synthetic sequences of the same length and the same maximal duplication length, created by the model described in [46, 47] with different μ/ν ratios. As shown in the figure, the μ/ν ratio is significantly heterogenous within and across genomes; different chromosomes of human and mouse show very different μ/ν ratios, varying from 0.001 (as mouse chromosome Y in subfigure (i)) to over 0.1 (as human chromosome 5 in subfigure (a)). Therefore, the μ/ν ratios selected for the numerical simulations exhibited in Fig. 5 of the main text (μ/ν = 0.001, 0.01, 0.1 and 1) should be typical and realistic for mammalian genomes. Figure S3. Standard deviations indicated by error bars for the Lastz net alignment exhibited in Fig. 5 of the main [file 12859_2020_3384_MOESM1_ESM.pdf]

Additional Files

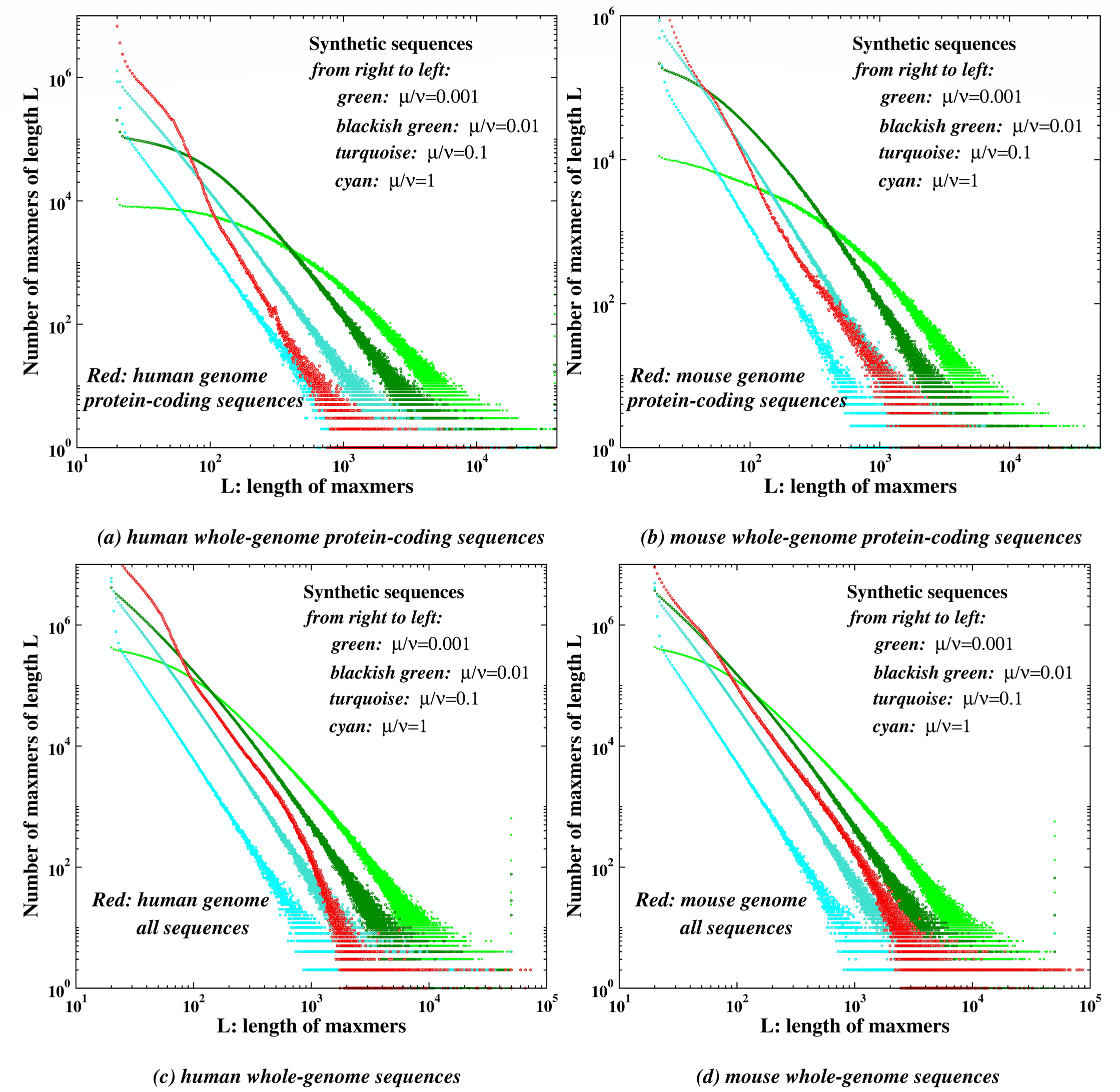

Figure S1. Match length distributions (MLDs) exhibited by histograms of maxmers for real genome sequences, as well as for synthetic sequences created by the model described in [46, 47]. Red curves in the figure exhibit the MLDs of the given real genome sequences, without any repeat- masking: (a) human whole-genome protein-coding sequences, (b) mouse whole-genome protein-coding sequences, (c) human whole-genome sequences, both protein-coding and non-protein-coding, and (d) mouse whole-genome sequences, both protein-coding and non-protein-coding. Other curves in the figure show MLDs for the synthetic sequences of the same length and the same maximal duplication length as the corresponding real genome sequence; different synthetic sequences are created with different μ/ν ratios. In each subfigure, by comparing the MLD of the real genome sequence to the MLDs of the synthetic sequences, we estimate the μ/ν ratio for the real genome sequence as following: (a) for human protein-coding genes, $\mu/\nu \approx 1$; (b) for mouse protein-coding genes $\mu/\nu \approx 0.1$; (c) and (d), for human and mouse whole-genome sequences, μ/ν is between 0.01 and 0.1.

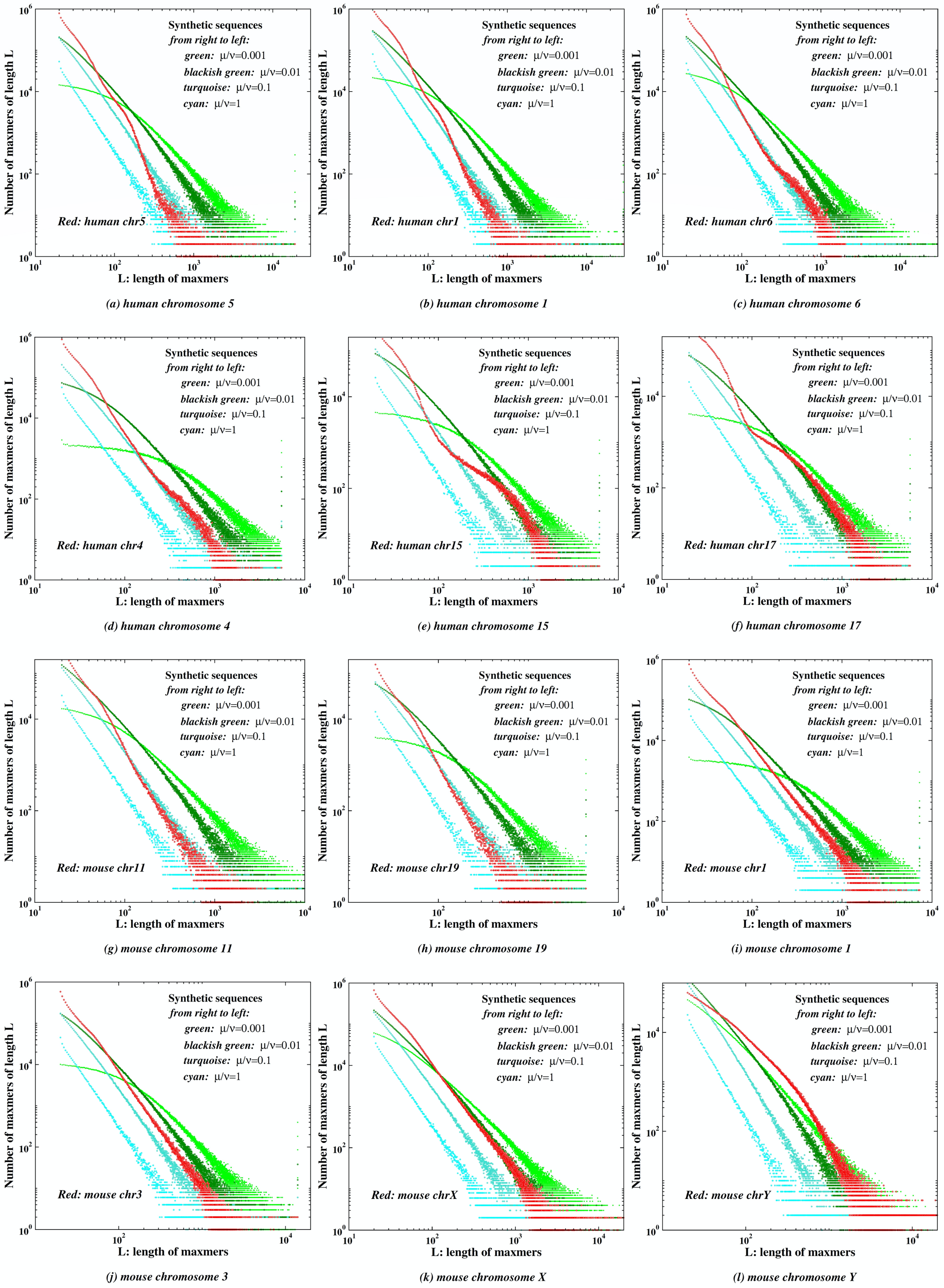

Figure S2. Match length distributions (MLDs) for different chromosomes of human and mouse, as well as for synthetic sequences of the same length and the same maximal duplication length, created by the model described in [46, 47] with different μ/ν ratios. As shown in the figure, the μ/ν ratio is significantly heterogenous within and across genomes; different chromosomes of human and mouse show very different μ/ν ratios, varying from 0.001 (as mouse chromosome Y in subfigure (i)) to over 0.1 (as human chromosome 5 in subfigure (a)). Therefore, the μ/ν ratios selected for the numerical simulations exhibited in Fig. 5 of the main text (μ/ν = 0.001, 0.01, 0.1 and 1) should be typical and realistic for mammalian genomes.

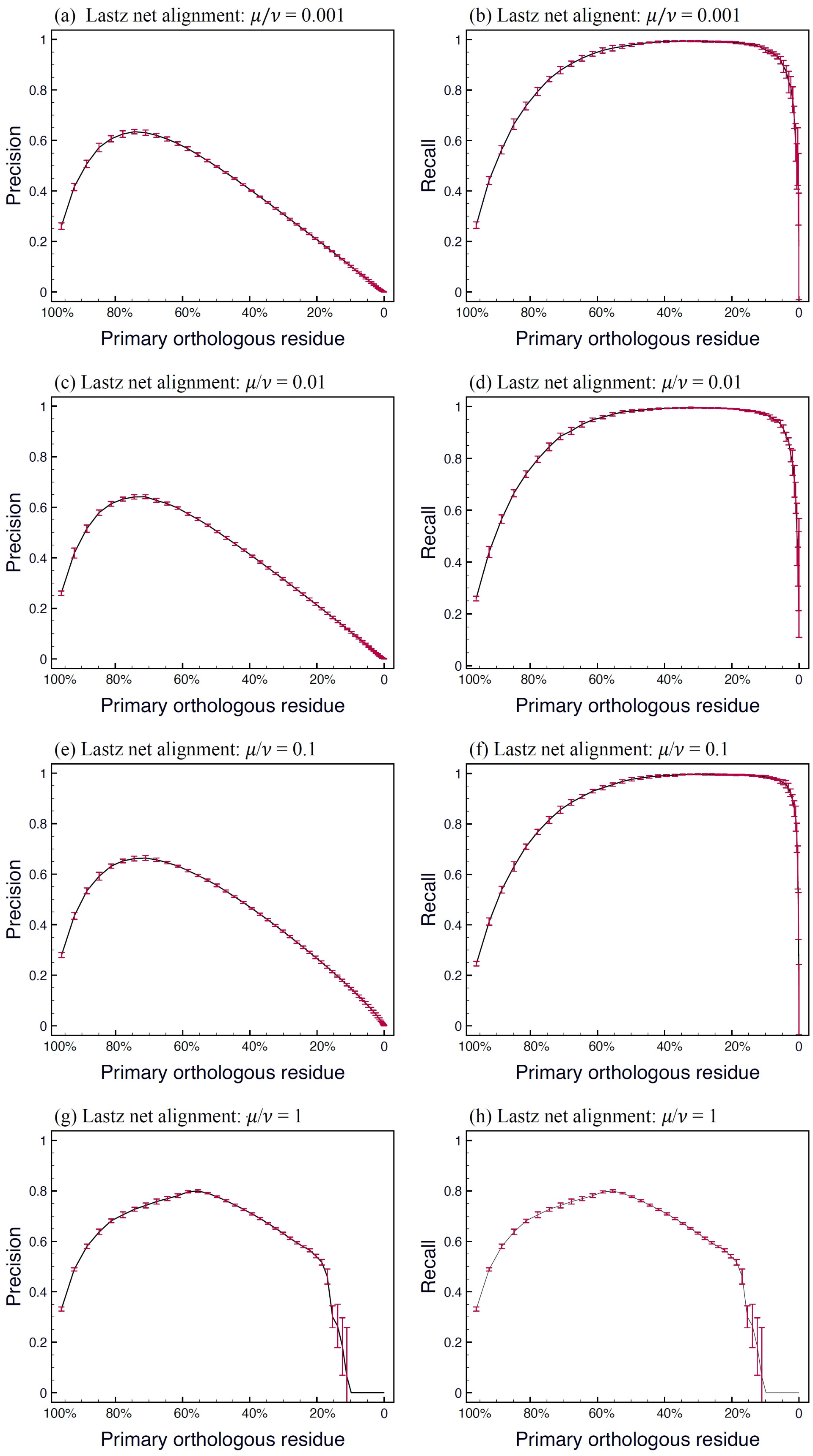

Figure S3. Standard deviations indicated by error bars for the Lastz net alignment exhibited in Fig. 5 of the main text.

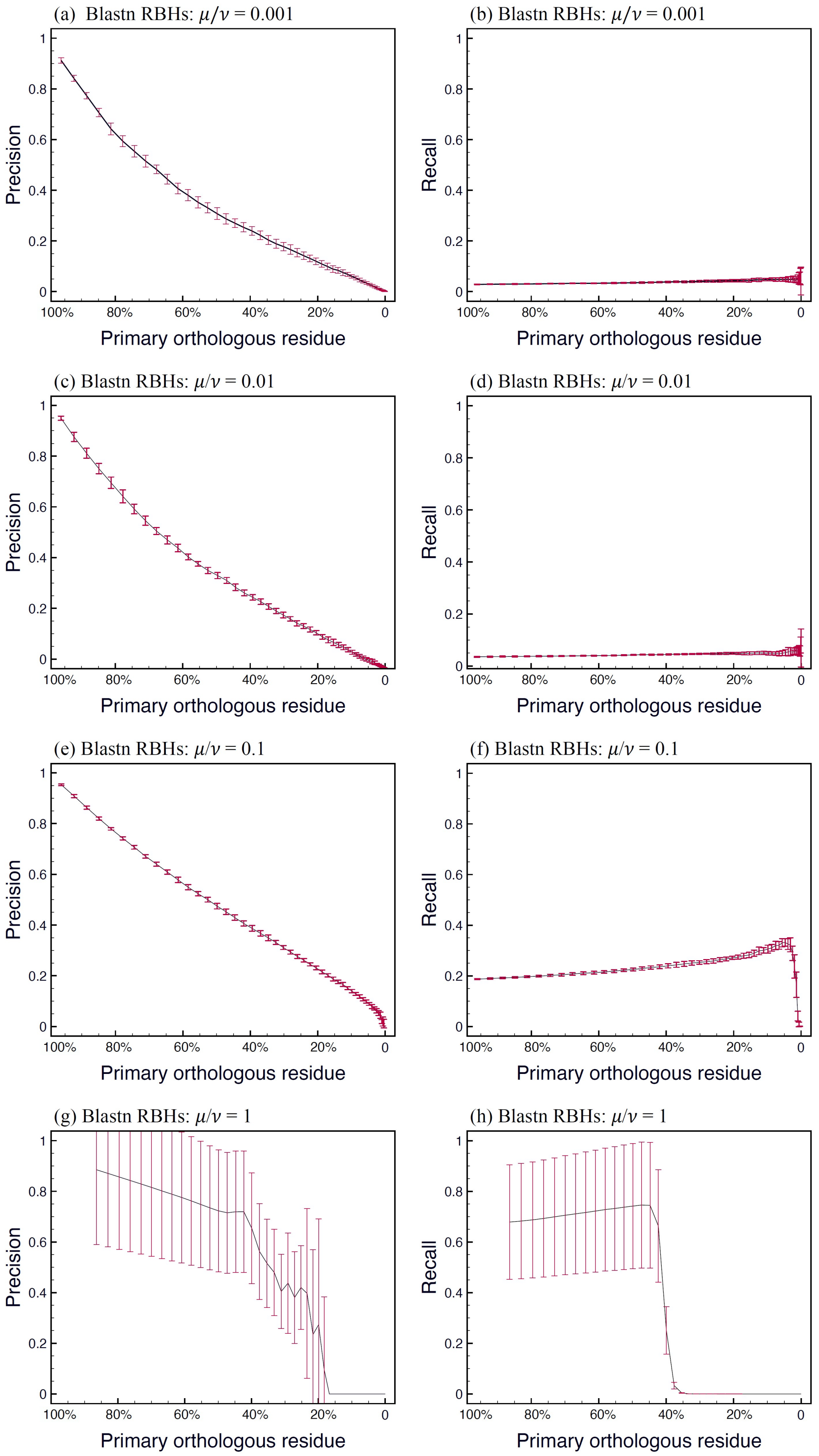

Figure S4. Standard deviations indicated by error bars for the BLAST RBHs exhibited in Fig. 5 of the main text.

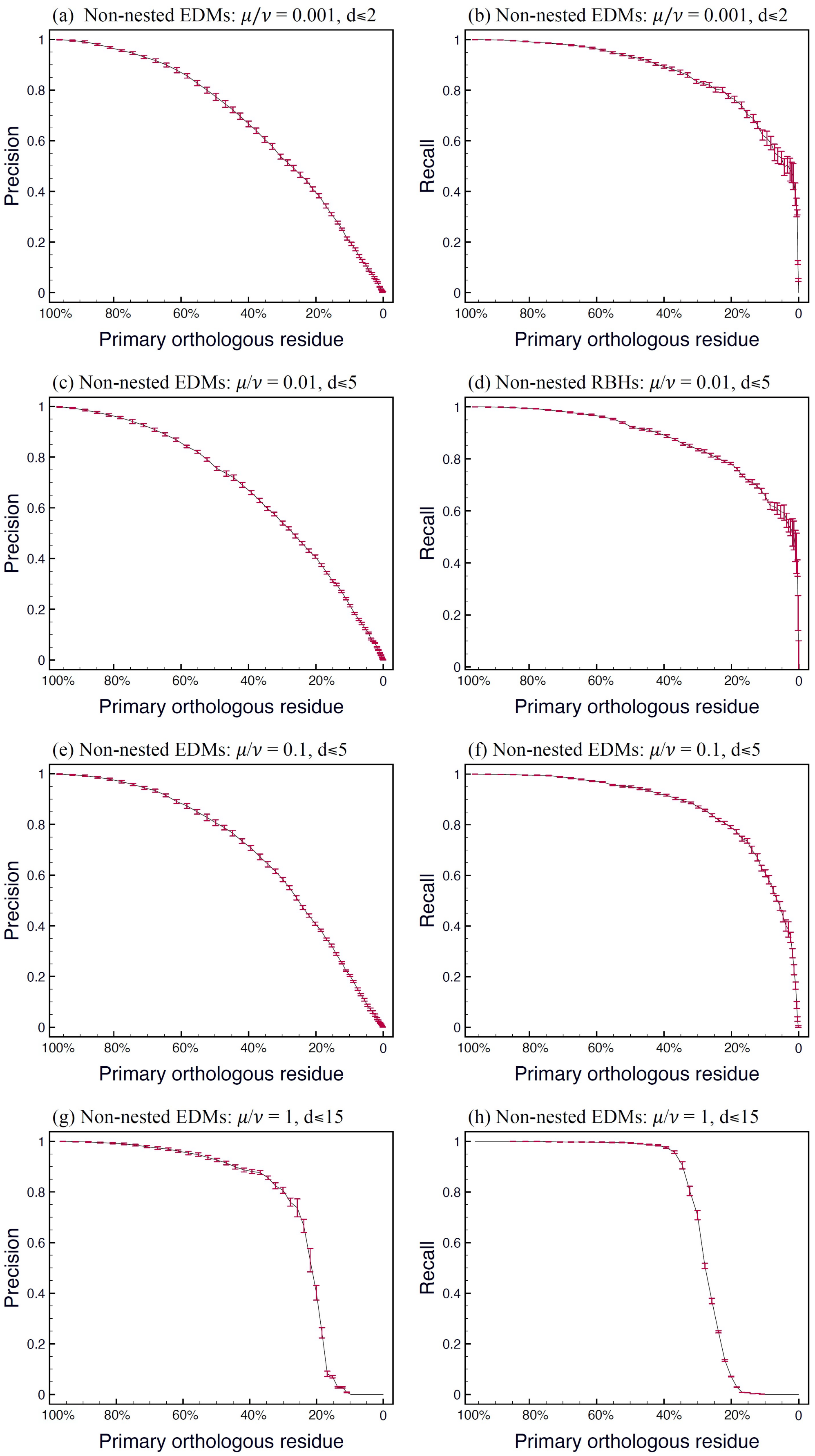

Figure S5. Standard deviations indicated by error bars for the non-nested EDMs exhibited in Fig. 5 of the main text.

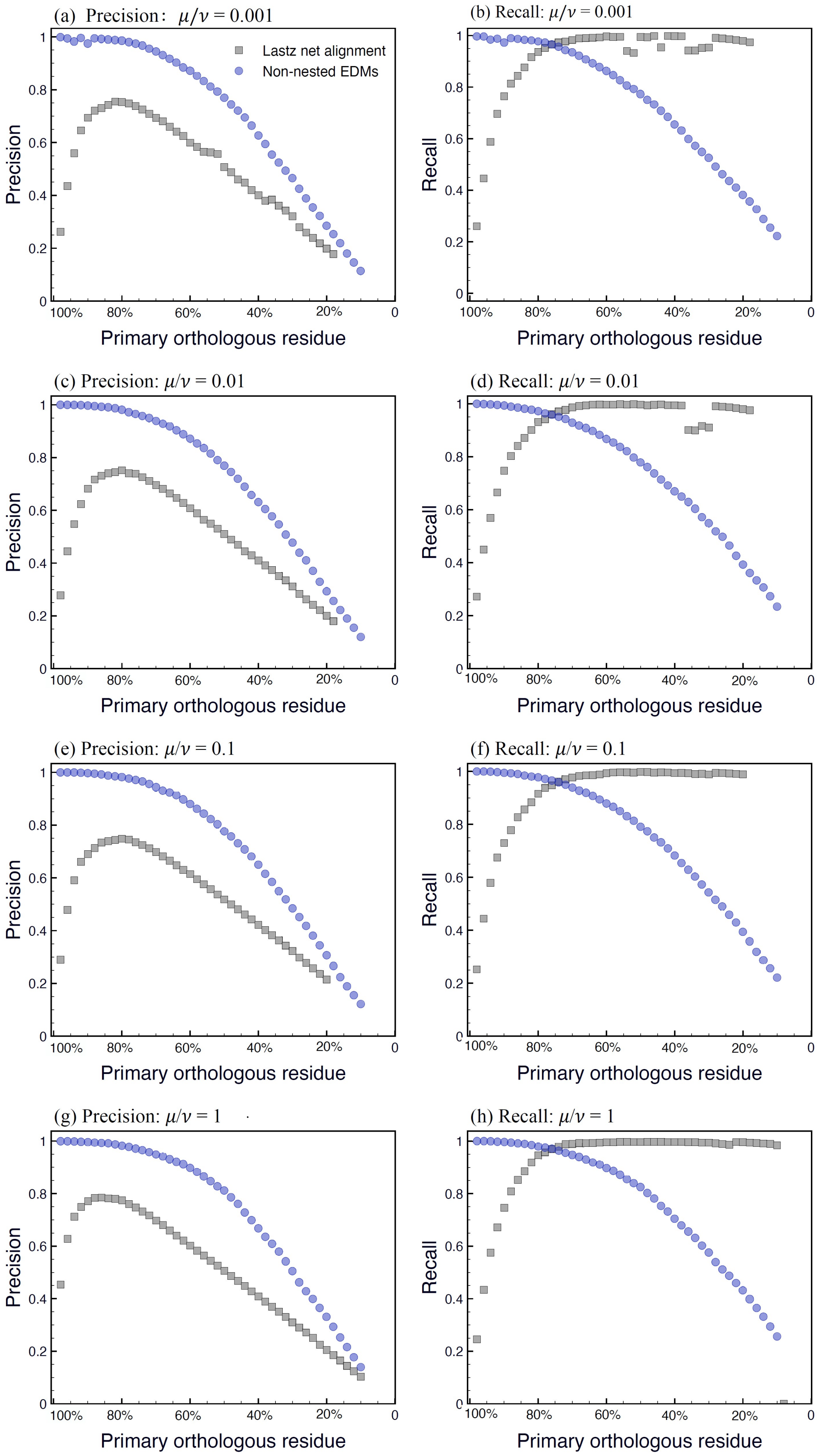

Figure S6. Precision and recall of our method derived from numerical simulations, in which each segmental duplication is inserted into the genome rather than substitutes another sequence of the same length in the same genome. Due to computation burden, we only simulate diverged lineages whose similarity is above 20% in a single realization. The result is essentially consistent with that in Fig. 5 of the main text.

Genome Pairs	Human & Gorilla	Human & Cat	Human & Chicken	Human & Anole lizard	Human & Frog	Human & Zebra fish	Human & Drosophila
**Total number of Non-nested RBHs**	26359	19449	6323	4754	2521	1617	475
**Precision *wrt*. Ensembl**	83% (21869)	83.6% (16254)	88.9% (5622)	83.9% (3989)	81.6% (2056)	63.8% (1031)	24.0% (114)
**Recall *wrt*. Ensembl**	95.1%	88.8%	41.5%	28.9%	15.7%	7.8%	2.3%
**Total number of BLAST RBHs**	24764	16151	6271	4878	2587	1597	206
**Precision *wrt*. Ensembl**	82.6% (20460)	83.3% (13449)	77.9% (4882)	74.5% (3636)	74.7% (1933)	57.3% (915)	29.6% (61)
**Recall *wrt*. Ensembl**	89%	73.5%	36.0%	26.3%	14.7%	6.9%	1.2%

Table S1. Statistics for figure S7, exhibiting the precision and recall of non-nested RBHs and BLAST RBHs, benchmarked by Ensembl CBGs. Numbers in the brackets indicate the numbers of RBHs shared between the corresponding sets.

Genome Pairs	$L_{\min}$	*P*	Numbers of mmRBHs	Precision 1	Precision 2	Recall 1	Recall 2
Human vs. Chimp	20	30.6%	7846	94.5% (7417)	66.2% (5193)	23.7% (5571)	27.3% (7039)
	30	86.3%	13271	95.6% (12687)	73.4% (9743)	44.4% (10446)	46.5% (11984)
	40	94.5%	23622	96.2% (22720)	78.2% (18479)	84.2% (19925)	82.6% (21274)
	50	97.0%	25346	95.7% (24261)	77.1% (19532)	89.6% (21213)	87.7% (22580)
	60	98.0%	25706	95.5% (24561)	76.6% (19701)	90.6% (21443)	88.6% (22819)
	70	98.5%	25716	95.3% (24498)	76.4% (19640)	90.5% (21399)	88.3% (22739)
	80	98.8%	25571	95.1% (24315)	76.4% (19524)	90.0% (21286)	87.6% (22553)
Human vs. Mouse	20	2.0%	1854	64.6% (1198)	36.1% (670)	5.5% (998)	6.0% (870)
	30	8.2%	2611	71.7% (1873)	51.4% (1341)	8.6% (1582)	11.2% (1632)
	40	31.2%	2443	77.0% (1880)	60.0% (1465)	9.3% (1698)	11.3% (1647)
	50	44.1%	2356	84.9% (2001)	68.3% (1609)	10.2% (1860)	12.0% (1750)
	60	61.9%	2429	88.9% (2159)	72.5% (1761)	11.0% (2020)	13.1% (1900)
	70	66.4%	2434	88.9% (2165)	73.4% (1786)	11.1% (2025)	13.2% (1926)
	80	69.7%	2305	88.8% (2046)	72.3% (1666)	10.4% (1904)	12.4% (1808)

Table S2. Performance of a "control experiment" in which mmRBHs are determined by *all* exact matches (both non-nested and nested). Parameters L_{min} refers to the minimal length of exact matches, and *P* refers to the proportion of hits on genes contributed by non-nested exact matches over those contributed by all exact matches, both weighted by lengths in bases. Precisions and recalls are defined as same as those in table 3 of the main text, except non-nested RBHs are substituted by mmRBHs determined by all exact matches: "Precision 1:" mmRBHs validated by *either* BLAST RBHs *or* Ensembl CBGs; "Precision 2:" mmRBHs validated by *both* BLAST RBHs *and* Ensembl CBGs; "Recall 1:" Ensembl CBGs recovered by mmRBHs; "Recall 2:" BLAST RBHs recovered by mmRBHs. Numbers in the brackets indicate the numbers of RBHs (or CBGs) shared by the corresponding sets. For human-chimpanzee, with large $L_{\min}$, non-nested exact matches overwhelmingly dominate nested ones in terms of hits on genes; mmRBHs determined by all exact matches and those solely by non-nested exact matches are nearly equivalent. For human-mouse, non-nested exact matches are not as dominant as for human-chimpanzee; therefore, for human-mouse, mmRBHs RBHs determined by all exact matches exhibit much lower precision and recall than those determined by non-nested exact matches.

Supplementary Material 1:
How to identify non-nested and nested maximal matches with *SEQANALYSIS*

SEQANALYSIS is a suffix array-based software package for quickly identifying certain classes of context-sensitive maxmers from pairs of eukaryote genome-length character sequences (Taillefer E and Miller J. Algebraic length-distribution of sequence duplications in whole genomes. In Proc of international conf on natural comput. Shanghai, China, Jul 2011; v3: 1454–1460; Taillefer E and Miller J. Exhaustive computation of exact sequence duplications in whole genomes via super and local maximal repeats. International Conf on Environ and Bio Sci (IPCBEE) IACSIT Press, Singapore. 2011; v21: 22–29). Source code can be obtained from (https://groups.oist.jp/sites/default/files/imce/u109/sequanalysis.zip).

To identify non-nested and nested maximal matches between two genomic sequences, the first step is to generate an “intersection.” *SEQANALYSIS* provides options that return in the output all *super*, *nested local* and *non-nested local* occurrences of maxmers (see (Taillefer E and Miller J. Exhaustive computation of exact duplications via super and non-nested local maximal repeats. J Bioinform Comput Biol. 2014; 12(1): 1350018) for details). We use the following command line for the intersections in this paper:

```
user@server:DIR> seqanalysis --mode=2SeqCountMtch --min-length=<minimal length of maxmers>
                 -L -P -p -fp -A -U -D -n -K -ao --ref-file=<fasta file for the reference sequence>
                 --query-file=<fasta file for the query sequence> --output-file=<output file>
```

where the options represent:

--mode=2SeqCountMtch	Pairwise intersection computation.
--list-match [-L]	List positions of all occurrences.
--print-matchseq [-P]	Print match strings.
--print-compact [-p]	List positions of all occurrences compactly.
--forward-pos [-fp]	For occurrence on the reverse strand, record the coordinate by its start position on the reverse strand, lead by a “-”. For example, -3 means the start position of an occurrence is at the third base of the reverse strand.
--max-match [-A]	Compute all maximal matches, irrespective of whether or not they are unique in either genome.
--uppercase [-U]	Pre-process the sequences by converting all alphabetical letters to uppercase.
--type-dna [-D]	Pre-process the sequences by converting all symbols other than {A, a, T, t, G, g, C, c, N, n} to “N” or “n”.
--cal-overlap [-n]	Output only super maxmers and non-nested occurrences of local maxmers.
--min-length=	Minimal match length.
--ref-file=	Input the reference sequence in fasta format. When the input file contains multiple sequences, *SEQANALYSIS* will preprocess these sequences by concatenating them all into a single long sequence, putting a “$” between neighboring

	sequences. Positions of maxmer occurrences in the output are reported relative to the single long sequence.
--query-file=	The same as *--ref-file* but for the query sequence.
--output-file=	Name of the output file.

Optional switches:

--append-revcomp [-K]	Compute intersections for both the forward strand and the reverse complement strand.
--print-allocc [-ao]	Print all maxmer occurrences (not only *super* and *non-nested local* but also *nested local*); -ao must be used together with –n.
--synpair [-sp]	Search and print the duplicated pairs of maxmer occurrences having equal distance occurrences (EDMs). The maxmers are identified from the pairwise-intersection of the input sequence. Below is an example of an EFI between two genome sequences GS-1 and GS-2, where the pair of maximal matches consists of the sub-sequences CTAC and GTTCC, having an inter-sequence spacing of d = 13. 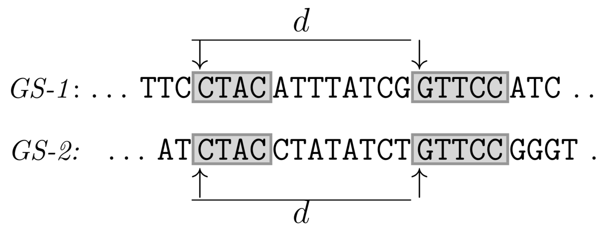

For more details, refer to *Manual.pdf* included in the source code of *SEQANALYSIS*.

The output file includes a list of all maxmer occurrences; non-nested and nested maximal matches can be obtained from the list of maxmer occurrences according to table 2 in the main text.

Section 1.1: When only non-nested maximal matches are needed

When only non-nested maximal matches are needed, we can turn off the switch *–ao* to ignore all occurrences of *nested local* in the intersection; this simplifies both the intersection and the identification of non-nested maximal matches. The output file reads like:

```
1   S:40608 114:AAAAAAAAAAAAAAAAAAAAAAAAAAAAAAAAAAAAAAAATTAATACATGATTTGATTTTAAATATTAAATGACTTTCTTTTT
    ATTTTCCTTCTCTTCTCTCTCACATTCTTAA 1:808567429 2:786292654
2   S:13896708 110:AAAAAAAAAAAATTAGCCGGGCATGGTGGCGGGCGCCTGTAGTCCCAGCTACTCGGGAGGCTGAGGCAGGAGAATGGCGT
    GAACCCGGGAGGCGGAGCTTGCAGTGAGCC 1:1069760770 1:-2726413043 2:205357112
3   L:38942624 101:AAAAAAATACGAAAACCAGTCAGGCGTGGCGGCGTGCGCCTGCAATCGCAGGCACTCGGCAGGCTGAGGCAGGAGAATCA
    GGCAGGGAGGTTGCAGTGAGC 1:1828466975 2:1340474564
4   L:806971 102:AAAAAAAAAAAAAAAAAAAAAAAAAGACTGTAGGAGCATCTGGTGGGAGGTGGTGGAGGGAGAACTGTGGGTTTGGAAGCTG
    CGCCCTCCCCCCAGCCATGC 2:2597025985
5   L:1004765 106:AAAAAAAAAAAAAAAAAAAAAAAAGAAGGAAGGAAGGGCCCAGAAGTCAGGAAGGAGCACGTGAGGAGGGTGTGTGGGAAG
    AATGGAGGTACTGAGGCAGGGTGCA 1:-2504991767
6   L:689564 110:AAAAAAAAAAAAAAAAAAAAAAAAAAGTCAGGAAACAACAGGTGCTGGAGAGGATGTGGAGAAATAGGAACACTTTTACACT
    GTTGGTGGGACTGTAAACTAGTTCAACC 1:-2897341951 1:-2290382390 1:-2216188336 1:-1822542408 2:3166049008
7   L:14377644758 172:GTTGTTGAATTTTGTCAAAGGCCTTTTCTGCATCTATTGAGATAATCATGTGGTTTTTGTCTTTGGTTCTGTTTATA
    TGCTGGATTACGTTTATTGATTTTCATATGTTGAACCAGCCTTGCATCCCAGGGATGAAGCCCACTTGATCATGGTGGATAAGCTTTTTGATGTG
    1:1135681928 2:-2709643878
```

Each line exhibits a maxmer. The left-most column shows the line numbers. The first column to the right indicates the maxmer type ("S" for super maxmer and "L" for local maxmer) and id # (a unique integer for each maxmer), separated by a colon; the second column indicates the length and the string of match, also separated by a colon; and the remaining columns indicate positions of all occurrences of *super* or *non-nested local* in both reference and query sequence ("1:" for occurrence in the reference sequence, and "2:" for occurrence in the query sequence; integers prefixed by "-" indicate that the corresponding occurrences appear in the reverse complement strand); columns are separated by spaces.

According to table 2 in the main text, for each maxmer appearing in the output file, since all these occurrences are either *super* or *non-nested local*, each occurrence in the reference sequence and each occurrence in the query sequence constitutes a non-nested maximal match; due to the same reason, every pair of occurrences must form a maximal match—there is no need to confirm this by checking their contexts.

In the sample output above, all lines except line 4 and 5 contribute non-nested maximal matches: line 4 contains no (non-nested) occurrence in the reference sequence, while line 5 contains no (non-nested) occurrence in the query sequence. Especially, line 1 contributes an *MUM*, since both occurrences are *unique supers*.

Section 1.2: When both non-nested and nested maximal matches are needed

When both non-nested and nested maximal matches are needed, we have to turn on the option *–ao* to include occurrences of *nested local* in the output file. The output file has a similar structure with the sample output in *S1.1*, except for its third column indicating four additional integers: the total number of occurrences in the reference sequence, the number of nested occurrences in the reference sequence, the total number of occurrences in the query sequence and the number of nested occurrences in the query sequence, separated by colons; from the fourth column on, positions of all occurrences (*super*, *non-nested local* and *nested local*) are listed.

```
1   S:40608 114:AAAAAAAAAAAAAAAAAAAAAAAAAAAAAAAAAAAAAAAATTAATACATGATTTGATTTTAAATATTAAATGACTTTCTTTTT
    ATTTTCCTTCTCTTCTCTCTCACATTCTTAA 1:0:1:0 1:808567429 2:786292654
2   S:13896708 110:AAAAAAAAAAAATTAGCCGGGCATGGTGGCGGGCGCCTGTAGTCCCAGCTACTCGGGAGGCTGAGGCAGGAGAATGGCGT
    GAACCCGGGAGGCGGAGCTTGCAGTGAGCC 2:0:1:0 1:1069760770 1:-2726413043 2:205357112
3   L:38942624 101:AAAAAAATACGAAAACCAGTCAGGCGTGGCGGCGTGCGCCTGCAATCGCAGGCACTCGGCAGGCTGAGGCAGGAGAATCA
    GGCAGGGAGGTTGCAGTGAGC 8:7:3:2 1:179419450 1:1828466975 1:2313972991 1:2349068155 1:-1274131725
    1:-1067359730 1:-983496574 1:-400874385 2:1304088511 2:1340474564 2:-489670408
4   L:806971 102:AAAAAAAAAAAAAAAAAAAAAAAAAGACTGTAGGAGCATCTGGTGGGAGGTGGTGGAGGGAGAACTGTGGGTTTGGAAGCTG
    CGCCCTCCCCCCAGCCATGC 2:2:3:2 1:2508659569 1:2534474142 2:2597025985 2:-2593711962 2:-2562502549
5   L:1004765 106:AAAAAAAAAAAAAAAAAAAAAAAAGAAGGAAGGAAGGGCCCAGAAGTCAGGAAGGAGCACGTGAGGAGGGTGTGTGGGAAG
    AATGGAGGTACTGAGGCAGGGTGCA 2:1:1:1 1:2499402921 1:-2504991767 2:2562500536
6   L:689564 110:AAAAAAAAAAAAAAAAAAAAAAAAAAGTCAGGAAACAACAGGTGCTGGAGAGGATGTGGAGAAATAGGAACACTTTTACACT
    GTTGGTGGGACTGTAAACTAGTTCAACC 4:0:1:0 1:-2897341951 1:-2290382390 1:-2216188336 1:-1822542408
    2:3166049008
7   L:14377644758 172:GTTGTTGAATTTTGTCAAAGGCCTTTTCTGCATCTATTGAGATAATCATGTGGTTTTTGTCTTTGGTTCTGTTTATA
    TGCTGGATTACGTTTATTGATTTTCATATGTTGAACCAGCCTTGCATCCCAGGGATGAAGCCCACTTGATCATGGTGGATAAGCTTTTTGATGTG
    3:2:3:2 1:1135681928 1:-916485511 1:-551977911 2:3096932611 2:-2709643878 2:-891973215
```

For super maxmers, the occurrences shown here are exactly the same to those shown in *S1.1*, and the inference of non-nested maximal matches from these occurrences is also the same. But for local maxmers, due to the existence of *nested local*, when inferring non-nested/nested maximal matches from the list of occurrences, **we need to check the contexts of each pair of occurrences to make sure they do form a maximal match**. The current version of *SEQANALYSIS* does not provide contexts of occurrences in the output file—we have to refer to the original sequences in the input files, and check out the immediate left and right contexts of each occurrence. For example, for line 7 in the above example output, we attach contexts to each occurrence:

```
7   L:14377644758 172:GTTGTTGAATTTTGTCAAAGGCCTTTTCTGCATCTATTGAGATAATCATGTGGTTTTTGTCTTTGGTTCTGTTTATA
    TGCTGGATTACGTTTATTGATTTTCATATGTTGAACCAGCCTTGCATCCCAGGGATGAAGCCCACTTGATCATGGTGGATAAGCTTTTTGATGTG
    3:2:3:2 1:1135681928:C:C 1:-916485511:G:A 1:-551977911:G:C 2:3096932611:G:T 2:-2709643878:T:T
    2:-891973215:A:A
```

We compare the contexts of each occurrence in the reference sequence to those of each occurrence in the query sequence; only when a pair of occurrences have different contexts on both sides, does it form a maximal match. Occurrences of local maxmer in the reference sequence that form maximal matches with every occurrence in the query sequence are non-nested locals, and *vice versa*. We discriminate all occurrences in line 7 as

**Compared sequences**	**Types of occurrence**	**Contexts** (left:right)	**positions of occurrences**
1 (reference)	*non-nested*	C:C	1135681928
	Nested	G:A	-916485511
		G:C	-551977911
2 (query)	*non-nested*	T:T	-2709643878
	Nested	G:T	3096932611
		A:A	-891973215

According to table 2 in the main text, these occurrences form the following maximal matches:

**Maximal matches**	**Combination of occurrences**	**Occurrence pairs**
Non-nested	*non-nested local* + *non-nested local*	1135681928:C:C *vs* -2709643878:T:T
Nested	*non-nested local* + *nested local*	1135681928:C:C *vs* 3096932611:G:T<br>1135681928:C:C *vs* -891973215:A:A<br>-916485511:G:A *vs* -2709643878:T:T<br>-551977911:G:C *vs* -2709643878:T:T
	nested local + *nested local*	-551977911:G:C *vs* -891973215:A:A

Supplementary Material 2

Ensembl annotations for human and chimpanzee genes that constitute non-nested RBHs but are *not* annotated as orthologs by Ensembl Compara.

Human Gene IDs	Human Gene Annotations	Chimp Gene IDs	Chimp Gene Annotations
ENSG00000187984	ankyrin repeat domain 19, pseudogene [Source:HGNC Symbol;Acc:HGNC:22567]	ENSPTRG00000048265	putative ankyrin repeat domain-containing protein 19-like [Source:NCBI gene;Acc:473343]
ENSG00000224227	olfactory receptor family 2 subfamily L member 1 pseudogene [Source:HGNC Symbol;Acc:HGNC:8265]	ENSPTRG00000048867	olfactory receptor 2L2-like [Source:NCBI gene;Acc:469756]
ENSG00000231649	SPATA31 subfamily B member 1, pseudogene [Source:HGNC Symbol;Acc:HGNC:31411]	ENSPTRG00000050795	spermatogenesis-associated protein 31C2-like [Source:NCBI gene;Acc:464935]
ENSG00000229117	ribosomal protein L41 [Source:HGNC Symbol;Acc:HGNC:10354]	ENSPTRG00000039769	ribosomal protein L41 [Source:NCBI gene;Acc:457859]
ENSG00000233056	endogenous retrovirus group 48 member 1 [Source:HGNC Symbol;Acc:HGNC:17216]	ENSPTRG00000051369	endogenous retrovirus group 48 member 1 [Source:NCBI gene;Acc:736930]
ENSG00000170967	DNA damage inducible 1 homolog 1 [Source:HGNC Symbol;Acc:HGNC:18961]	ENSPTRG00000028414	DNA damage inducible 1 homolog 1 [Source:NCBI gene;Acc:741052]
ENSG00000229230	metallothionein 1 pseudogene 3 [Source:HGNC Symbol;Acc:HGNC:16120]	ENSPTRG00000044049	metallothionein-1L [Source:NCBI gene;Acc:101058101]
ENSG00000170688	olfactory receptor family 5 subfamily E member 1 pseudogene [Source:HGNC Symbol;Acc:HGNC:8342]	ENSPTRG00000045607	olfactory receptor 1044-like [Source:NCBI gene;Acc:466432]
ENSG00000188873	ribosomal protein L10a pseudogene 2 [Source:HGNC Symbol;Acc:HGNC:31081]	ENSPTRG00000042887	60S ribosomal protein L10a-like [Source:NCBI gene;Acc:464158]
ENSG00000176294	olfactory receptor family 4 subfamily N member 2 [Source:NCBI gene;Acc:390429]	ENSPTRG00000006075	olfactory receptor 4N2 [Source:NCBI gene;Acc:465195]
ENSG00000207925	microRNA 516b-2 [Source:HGNC Symbol;Acc:HGNC:32117]	ENSPTRG00000047860	ptr-mir-516b-2 [Source:miRBase;Acc:MI0008708]
ENSG00000259158	ADAM metallopeptidase domain 20 pseudogene 1 [Source:HGNC Symbol;Acc:HGNC:20102]	ENSPTRG00000051900	disintegrin and metalloproteinase domain-containing protein 20-like [Source:NCBI gene;Acc:467495]
ENSG00000188662	histone linker H1 domain, spermatid-specific 1 (pseudogene) [Source:HGNC Symbol;Acc:HGNC:30616]	ENSPTRG00000046816	spermatid-specific linker histone H1-like protein [Source:NCBI gene;Acc:100612812]
ENSG00000207573	microRNA 550a-2 [Source:HGNC Symbol;Acc:HGNC:32805]	ENSPTRG00000050734	ptr-mir-550-3 [Source:miRBase;Acc:MI0008767]
ENSG00000185385	olfactory receptor family 7 subfamily A member 17 [Source:HGNC Symbol;Acc:HGNC:8363]	ENSPTRG00000029099	olfactory receptor 7A17 [Source:NCBI gene;Acc:747846]

ENSG00000257137	chromosome 12 open reading frame 80 [Source:HGNC Symbol;Acc:HGNC:27473]	ENSPTRG00000053807	chromosome 12 C12orf80 homolog [Source:NCBI gene;Acc:100608471]
ENSG00000256779	serine/arginine-rich splicing factor 8 (SRSF8) pseudogene	ENSPTRG00000043643	serine/arginine-rich splicing factor 8-like [Source:NCBI gene;Acc:738374]
ENSG00000204571	keratin associated protein 5-11 [Source:HGNC Symbol;Acc:HGNC:23606]	ENSPTRG00000028491	keratin associated protein 5-11 [Source:VGNC Symbol;Acc:VGNC:3112]
ENSG00000235812	ADAM metallopeptidase domain 21 pseudogene 1 [Source:HGNC Symbol;Acc:HGNC:19822]	ENSPTRG00000006493	ADAM metallopeptidase domain 21 [Source:VGNC Symbol;Acc:VGNC:12959]
ENSG00000174125	toll like receptor 1 [Source:HGNC Symbol;Acc:HGNC:11847]	ENSPTRG00000015985	toll like receptor 1 [Source:VGNC Symbol;Acc:VGNC:12857]
ENSG00000227067	developmental pluripotency associated 3 pseudogene 1 [Source:HGNC Symbol;Acc:HGNC:32345]	ENSPTRG00000042080	developmental pluripotency-associated protein 3 [Source:NCBI gene;Acc:737717]
ENSG00000273769	lung carcinoma-associated protein (LCAP)	ENSPTRG00000039921	putative lung carcinoma-associated protein 10 [Source:NCBI gene;Acc:101057533]
ENSG00000234287	ribosomal protein S27 (RPS27) pseudogene	ENSPTRG00000046188	40S ribosomal protein S27-like [Source:NCBI gene;Acc:739591]
ENSG00000213005	pituitary tumor-transforming 3, pseudogene [Source:HGNC Symbol;Acc:HGNC:13422]	ENSPTRG00000052460	Putative pituitary tumor-transforming gene 3 protein [Source:UniProtKB/Swiss-Prot;Acc:Q2QD15]
ENSG00000225051	high mobility group box 3 pseudogene 22 [Source:HGNC Symbol;Acc:HGNC:39314]	ENSPTRG00000051338	high mobility group protein B3-like [Source:NCBI gene;Acc:471771]
ENSG00000204464	chromosome 1 open reading frame 195 [Source:HGNC Symbol;Acc:HGNC:32332]	ENSPTRG00000030816	chromosome 1 C1orf195 homolog [Source:NCBI gene;Acc:104006312]
ENSG00000147403	ribosomal protein L10 [Source:HGNC Symbol;Acc:HGNC:10298]	ENSPTRG00000043378	ribosomal protein L10 [Source:NCBI gene;Acc:465944]
ENSG00000122872	ADP ribosylation factor like GTPase 4A pseudogene 1 [Source:HGNC Symbol;Acc:HGNC:17741]	ENSPTRG00000052279	ADP-ribosylation factor-like protein 4A [Source:NCBI gene;Acc:450480]
ENSG00000174130	toll like receptor 6 [Source:HGNC Symbol;Acc:HGNC:16711]	ENSPTRG00000051210	toll like receptor 6 [Source:NCBI gene;Acc:471167]
ENSG00000071082	ribosomal protein L31 [Source:HGNC Symbol;Acc:HGNC:10334]	ENSPTRG00000050218	ribosomal protein L31 [Source:NCBI gene;Acc:743643]
ENSG00000185088	ribosomal protein S27 like [Source:HGNC Symbol;Acc:HGNC:18476]	ENSPTRG00000007144	ribosomal protein S27 like [Source:NCBI gene;Acc:100612661]
ENSG00000226098	SEC11 homolog B, signal peptidase complex subunit (pseudogene) [Source:HGNC Symbol;Acc:HGNC:31884]	ENSPTRG00000044848	putative signal peptidase complex catalytic subunit SEC11B [Source:NCBI gene;Acc:464179]
ENSG00000263781	NADH dehydrogenase (ubiquinone) Fe-S protein 5, 15kDa (NADH-coenzyme Q reductase) (NDUFS5) pseudogene	ENSPTRG00000049323	NADH dehydrogenase [ubiquinone] iron-sulfur protein 5-like [Source:NCBI gene;Acc:107969209]

ENSG00000177233	olfactory receptor family 2 subfamily M member 1 pseudogene [Source:HGNC Symbol;Acc:HGNC:8267]	ENSPTRG00000049161	olfactory receptor 2M3-like [Source:NCBI gene;Acc:739832]
ENSG00000169474	small proline rich protein 1A [Source:HGNC Symbol;Acc:HGNC:11259]	ENSPTRG00000030381	small proline rich protein 1A [Source:VGNC Symbol;Acc:VGNC:10966]
ENSG00000230453	ankyrin repeat domain 18B [Source:HGNC Symbol;Acc:HGNC:23644]	ENSPTRG00000020960	ankyrin repeat domain 18B [Source:VGNC Symbol;Acc:VGNC:4613]
ENSG00000224837	glycine cleavage system protein H pseudogene 5 [Source:HGNC Symbol;Acc:HGNC:44195]	ENSPTRG00000051719	glycine cleavage system H protein, mitochondrial [Source:NCBI gene;Acc:742399]
ENSG00000180747	SMG1 pseudogene 3 [Source:HGNC Symbol;Acc:HGNC:49860]	ENSPTRG00000033847	serine/threonine-protein kinase SMG1-like [Source:NCBI gene;Acc:749371]
ENSG00000236382	keratin associated protein 10-13, pseudogene [Source:HGNC Symbol;Acc:HGNC:34213]	ENSPTRG00000040577	keratin-associated protein 10-3 [Source:NCBI gene;Acc:738736]
ENSG00000257074	ribosomal protein L29 pseudogene 33 [Source:HGNC Symbol;Acc:HGNC:36923]	ENSPTRG00000050033	60S ribosomal protein L29-like [Source:NCBI gene;Acc:104003347]
ENSG00000251247	zinc finger protein 345 [Source:HGNC Symbol;Acc:HGNC:16367]	ENSPTRG00000022702	zinc finger protein 345 [Source:NCBI gene;Acc:100615948]
ENSG00000242970	ribosomal protein S26 (RPS26) pseudogene	ENSPTRG00000050850	40S ribosomal protein S26-like [Source:NCBI gene;Acc:464350]
ENSG00000163467	TSSK6 activating cochaperone [Source:HGNC Symbol;Acc:HGNC:30636]	ENSPTRG00000001457	TSSK6 activating cochaperone [Source:VGNC Symbol;Acc:VGNC:7018]
ENSG00000249244	glycerol kinase 2 pseudogene	ENSPTRG00000023546	glycerol kinase 2 [Source:VGNC Symbol;Acc:VGNC:10864]
ENSG00000273586	olfactory receptor family 10 subfamily G member 1 pseudogene [Source:HGNC Symbol;Acc:HGNC:8169]	ENSPTRG00000048781	olfactory receptor 10G2 [Source:NCBI gene;Acc:742615]
ENSG00000155918	retinoic acid early transcript 1L [Source:HGNC Symbol;Acc:HGNC:16798]	ENSPTRG00000044735	retinoic acid early transcript 1L [Source:NCBI gene;Acc:463159]
ENSG00000254769	olfactory receptor family 4 subfamily A member 4 pseudogene [Source:HGNC Symbol;Acc:HGNC:15161]	ENSPTRG00000043012	olfactory receptor 4A47-like [Source:NCBI gene;Acc:746547]
ENSG00000170468	ribosomal oxygenase 1 [Source:HGNC Symbol;Acc:HGNC:20968]	ENSPTRG00000039634	ribosomal oxygenase 1 [Source:VGNC Symbol;Acc:VGNC:3468]
ENSG00000224236	mitochondrial ribosome recycling factor pseudogene 1 [Source:HGNC Symbol;Acc:HGNC:35238]	ENSPTRG00000041636	ribosome-recycling factor, mitochondrial-like [Source:NCBI gene;Acc:465841]
ENSG00000267418	elongin C pseudogene 29 [Source:HGNC Symbol;Acc:HGNC:49170]	ENSPTRG00000010240	elongin-C-like [Source:NCBI gene;Acc:744141]
ENSG00000256681	coiled-coil domain containing 58 pseudogene 5 [Source:HGNC Symbol;Acc:HGNC:45028]	ENSPTRG00000045488	coiled-coil domain-containing protein 58-like [Source:NCBI gene;Acc:100609546]

ENSG00000233718	MYCN opposite strand [Source:HGNC Symbol;Acc:HGNC:16911]	ENSPTRG00000042767	MYCN opposite strand [Source:NCBI gene;Acc:100609397]
ENSG00000164616	F-box and leucine rich repeat protein 21, pseudogene [Source:HGNC Symbol;Acc:HGNC:13600]	ENSPTRG00000044075	F-box and leucine rich repeat protein 21, pseudogene [Source:NCBI gene;Acc:471642]
ENSG00000237550	ribosomal protein L9 pseudogene 9 [Source:HGNC Symbol;Acc:HGNC:17251]	ENSPTRG00000049459	60S ribosomal protein L9-like [Source:NCBI gene;Acc:465956]
ENSG00000227877	myoregulin [Source:HGNC Symbol;Acc:HGNC:48649]	ENSPTRG00000045653	myoregulin [Source:NCBI gene;Acc:100612984]
ENSG00000214896	ribosomal protein SA pseudogene 55 [Source:HGNC Symbol;Acc:HGNC:36921]	ENSPTRG00000048408	40S ribosomal protein SA-like [Source:NCBI gene;Acc:107968518]
ENSG00000236683	high mobility group AT-hook 1 pseudogene 1 [Source:HGNC Symbol;Acc:HGNC:13321]	ENSPTRG00000051684	high mobility group protein HMG-I/HMG-Y-like [Source:NCBI gene;Acc:101057227]
ENSG00000230997	RAB42, member RAS oncogene family, pseudogene 1 [Source:HGNC Symbol;Acc:HGNC:19799]	ENSPTRG00000039742	ras-related protein Rab-42-like [Source:NCBI gene;Acc:737297]
ENSG00000207861	microRNA 520h [Source:HGNC Symbol;Acc:HGNC:32125]	ENSPTRG00000043023	ptr-mir-520g [Source:miRBase;Acc:MI0008729]
ENSG00000260602	high mobility group nucleosomal binding domain 2 pseudogene 40 [Source:HGNC Symbol;Acc:HGNC:39411]	ENSPTRG00000050555	non-histone chromosomal protein HMG-17 [Source:NCBI gene;Acc:738529]
ENSG00000231551	phosphodiesterase 4D interacting protein (PDE4DIP) pseudogene	ENSPTRG00000045351	neuroblastoma breakpoint family member 6-like protein [Source:NCBI gene;Acc:748981]
ENSG00000197982	chromosome 1 open reading frame 122 [Source:HGNC Symbol;Acc:HGNC:24789]	ENSPTRG00000000558	chromosome 1 C1orf122 homolog [Source:NCBI gene;Acc:456765]
ENSG00000235616	ST13, Hsp70 interacting protein pseudogene 2 [Source:HGNC Symbol;Acc:HGNC:38716]	ENSPTRG00000045136	hsc70-interacting protein-like [Source:NCBI gene;Acc:459811]
ENSG00000223731	SUPT20H like 1 [Source:HGNC Symbol;Acc:HGNC:30773]	ENSPTRG00000045217	transcription factor SPT20 homolog-like 1 [Source:NCBI gene;Acc:100610514]

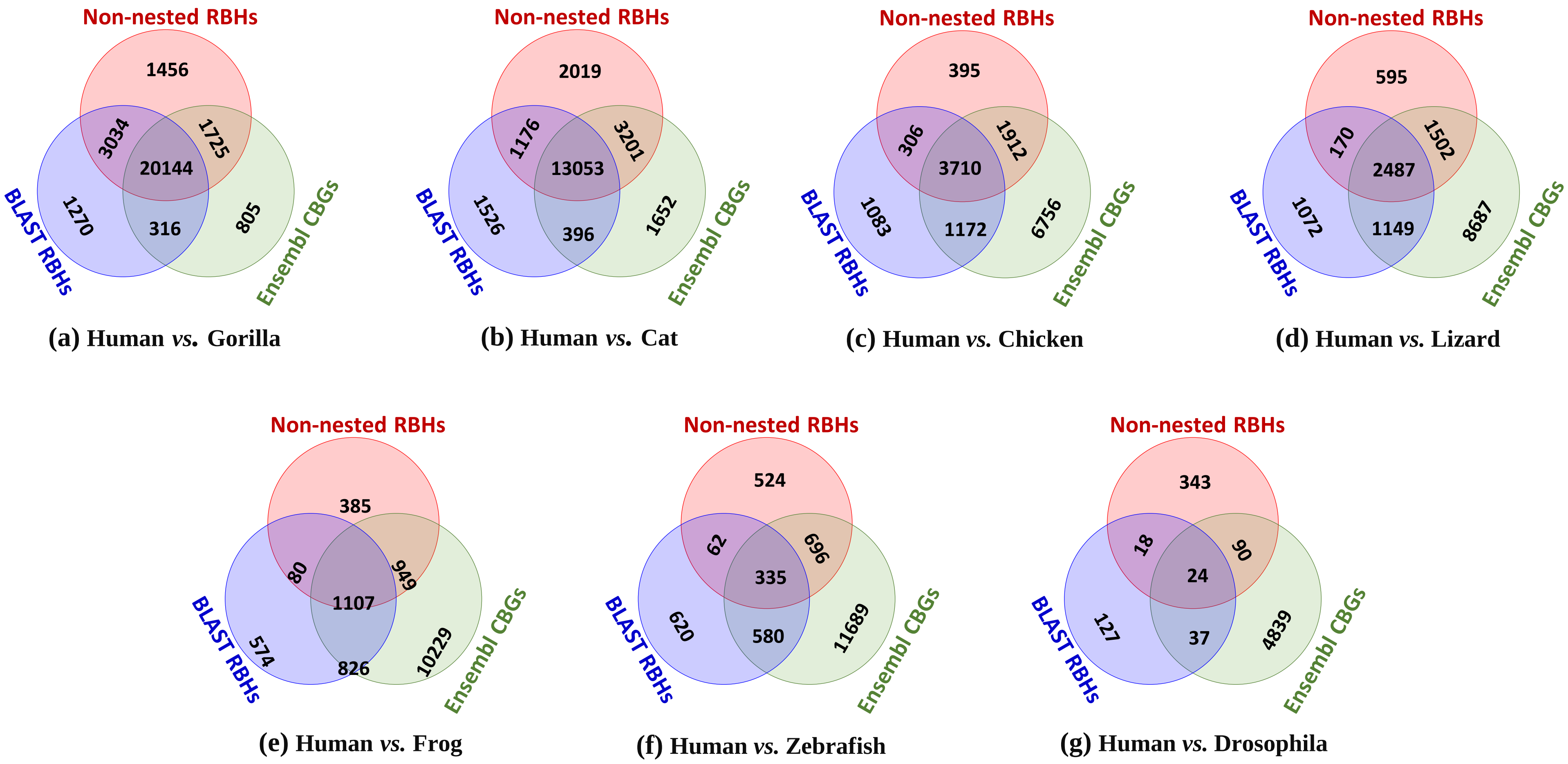

Figure S7. Venn diagrams among non-nested RBHs, BLAST RBHs and Ensembl CBGs between the genomes of human against four different species: (a) gorilla (*Gorilla gorilla*), (b) cat (*Felis catus*), (c) chicken (*Gallus gallus*), (d) anole lizard (*Anolis carolinensis*), (e) frog (*Xenopus tropicalis*), (f) zebrafish (*Danio rerio*), and (g) drosophila (*Drosophila melanogaster*). From (a) to (g), species are more and more distantly related to human; in this figure we use *MUMs* longer than 15 nucleotide bases to determine the non-nested RBHs. Estimated by the proportion of non-nested RBHs validated by *either* BLAST RBHs *or* Ensembl CBGs, the precision of our method in each subfigure is: (a) 94.5%, (b) 89.6%, (c) 93.8%, (d) 87.5%, (e) 84.7%, (f) 67.6% and (g) 27.8%.
